# QTAIM Analysis of a [2]Rotaxane Molecular Shuttle with a 2,2′‐Bipyridyl Rigid Core

**DOI:** 10.1002/cphc.202500074

**Published:** 2025-04-21

**Authors:** Costantino Zazza, Nico Sanna, Stefano Borocci, Felice Grandinetti

**Affiliations:** ^1^ Department for Innovation in Biological Agro‐food and Forest Systems Università della Tuscia (DIBAF) L.go dell’Università, s.n.c. 01100 Viterbo Italy; ^2^ Istituto per la Scienza e Tecnologia dei Plasmi del CNR (ISTP) Via G. Amendola 122/D 70126 Bari Italy; ^3^ Istituto per i Sistemi Biologici del CNR (ISB) Sede di Roma ‐ Meccanismi di Reazione c/o Dipartimento di Chimica Sapienza Università di Roma P.le A. Moro 5 Rome Italy

**Keywords:** density functional theory, molecular shuttles, nanoscale devices, quantum theory of atoms in molecules

## Abstract

Chemical contacts responsible for the supramolecular assembly of a rigid H‐shaped [2]rotaxane molecular shuttle composed of a 24‐crown‐8(**24C8**) macrocycle on a molecular thread containing two benzimidazole (Bzi) recognition sites and a central 2,2′‐bipyridyl (Bipy) rigid core are analytically addressed by combining the Quantum Theory of Atoms in Molecules (QTAIM) with density functional theory (DFT). In this respect, the available crystallographic structure—CCDC number 2248267—is taken as a reference condition for addressing the nature of the chemical interactions finely modulating the shuttling of the **24C8** between Bzi stations. Moreover, previous DFT computations (*Chem. Sci.*, **2023**, *14*, 7215) are extended over a supercomputing environment to address the proposed ligand exchange mechanism involving DMF solvent molecules and promoting the observed shuttling process upon the addition of Zn(II) cations. To this end, converged DFT wavefunctions are fully analyzed by means of electron density *ρ(r)* and local electronic energy density—*H(r)*—descriptors; interestingly, the derived covalent versus noncovalent interaction patterns shed some light on the mutual position of the macrocycle along the axle following the coordination of Zn(II) ions in DMF solvent.

## Graphical abstract

Supramolecular assembly of a rigid H‐shaped [2]rotaxane composed of 24C8 macrocycle on a molecular thread containing two benzimidazole recognition sites and a central 2,2’‐bipyridyl core is investigated by combining QTAIM with DFT algorithms. The derived interaction patterns shed some light on the mutual position of the 24C8 ring along the axle following the coordination of Zn(II) ions in DMF solvent.

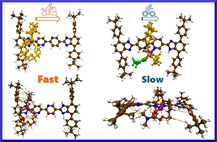

## 1 Introduction

Molecular rotaxanes represent a fascinating class of mechanically interlocked molecules (MIMs), distinguished by their unique architecture where a linear molecular component (the “axle”) is threaded through a macrocyclic ring (the “wheel”), with bulky end groups preventing disassembly.^[^ ^1^, ^2^, ^3^ ^]^ This structure, which is reminiscent of the components of a traditional axle and wheel, has inspired significant interest in the field of supramolecular chemistry due to its potential applications in molecular machines, drug delivery systems, and advanced materials.^[^ ^4^, ^5^, ^6^ ^]^ The concept of MIMs like rotaxanes challenges the traditional understanding of chemical bonding, as the components are held together not by traditional covalent bonds but through supramolecular forces modulating the conformational shaping in different contexts. This distinctive feature allows for novel functions and behaviors that are not possible in covalently bonded systems.^[^ ^7^, ^8^, ^9^ ^]^ The synthesis of rotaxanes has evolved from serendipitous discoveries to more sophisticated templating strategies, allowing for greater control over the size, shape, and functionality of the supramolecular aggregates involved. Recent advances in the field have led to the development of stimuli‐responsive rotaxanes, which can change their conformation or undergo quasimonodimensional molecular shuttling in response to external stimuli such as light, pH, ion addition, or redox conditions.^[^ ^10^, ^11^, ^12^, ^13^, ^14^, ^15^, ^16^, ^17^, ^18^, ^19^ ^]^ These fascinating responsive dynamic properties have opened up new avenues for research, particularly in the creation of molecular switches, sensors, and motors at the nanoscale level.^[^ ^20^ ^]^ Moreover, large amplitude translation of macrocycles in rotaxanes has made them promising candidates for biomedical applications, including drug delivery and molecular recognition systems. In this respect, Loeb and coworkers have recently proposed a fascinating interlocked H‐shaped [2]molecular rotaxane composed by a 24‐crown‐8 ether (**24C8**) macrocycle in supramolecular interaction with a molecular thread featuring a central 2,2′‐bipyridyl (Bipy) core and two symmetrical benzimidazole (Bzi) recognition sites.^[^ ^21^ ^]^ The central chemical unit was found to act as a speed bump raising the barrier to **24C8** shuttling movement between the two Bzi moieties as a result of the electronic repulsion involving Bipy N‐atoms and the crown ether *O*‐atoms.^[^ ^22^ ^]^ Moreover, the addition of Zn(II) ions in a coordinating solvent like the N,N‐dimethylformamide (DMF) promoted the shuttling of the **24C8** via a ligand exchange mechanism most likely occurring by coordination of the macrocycle itself to an intermediate compound in which the metal ion is bound to the Bipy chelate.^[^ ^21^ ^]^ Herein, we fully addressed supramolecular contacts between the **24C8** and the linear molecular thread via the Quantum Theory of Atoms in Molecules (QTAIM)^[^ ^23^, ^24^, ^25^ ^]^ in the framework of the density functional theory (DFT).^[^ ^26^ ^]^ In particular, previous DFT computations carried out by Loeb and coworkers^[^ ^21^ ^]^ are extended in accuracy running simulations over a supercomputing facility in conjunction with electron density *ρ(r)*, local electronic energy density *H(r)*, and Bader's topology analysis descriptors to obtain detailed information regarding the nature of the interactions supporting the existence of the proposed transient species. All our computations strongly support the existence of the hypothesized solvent/macrocycle exchange mechanism in the investigated rotaxane containing a chelating Bipy unit for Zn(II) cations.

## 2 Results and Discussion

### 2.1 Theoretical Characterization of the [2]Rotaxane Supramolecular Assembly

**Figure 1:**
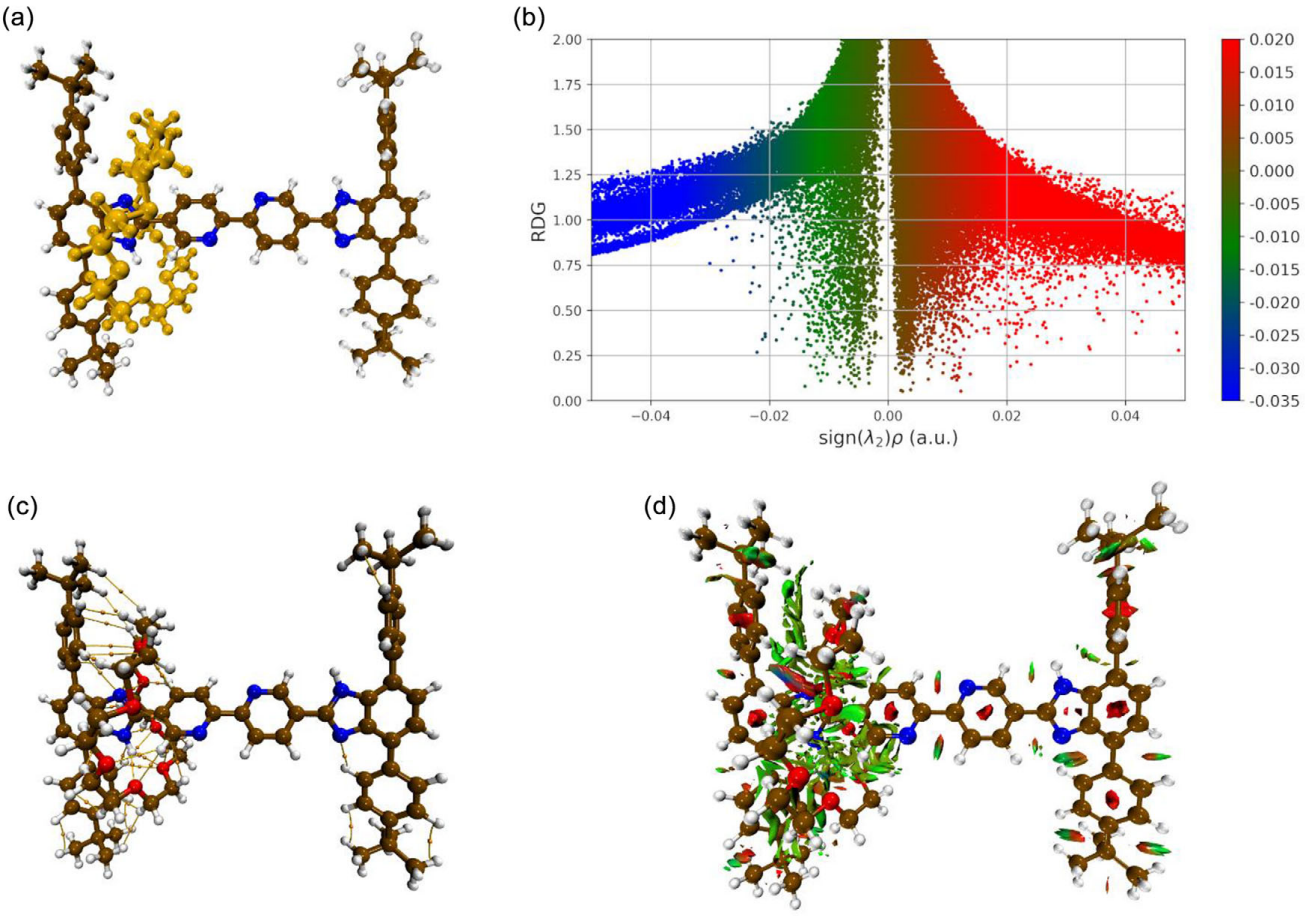
a) Molecular structure of the investigated molecular shuttle (CCDC: 2248267); the **24C8** macrocycle is reported in orange. b) The related 2D RDG plots in which the color bar represents the sign(*λ*_2_ *) × ρ(r)* in atomic units. c) Noncovalent bond paths (BPs)—at B3LYP(D3)/cc‐pVTZ level of theory—connecting the **24C8** with the Bzi, Bipy, and stopper moieties; the BPs are those analyzed in Table 1. d) 3D plot of NCIs characterizing supramolecular interaction patterns in the X‐ray structure; blue refers to strong electrostatic attractions, green to vdW (either attractive or repulsive) interactions, and red to strong electrostatic repulsions.

We first investigated the nature of van der Waals (vdWs) interactions, hydrogen bonds, and steric repulsion effects characterizing the supramolecular structure derived in the X‐Ray structure of the investigated [2]rotaxane and shown in **Figure** **1**a. The same figure displays the analyses carried out for addressing the nature of the host(the thread):guest(the macrocycle) contacts at B3LYP(D3)/cc‐pVZT level of theory. The 2D plot of the RDG versus sign(*λ*_2_ *) × ρ(r)* in Figure 1b allows to characterize noncovalent attractive interactions signaled by the QTAIM analysis in Figure 1c and summarized in **Table** **1**. The RDG surface in blue indicates stronger supramolecular interactions like hydrogen bonding contacts between the amino group of the Bzi site and the ether oxygen atoms in the **24C8** ring (see Figure 1d); green regions can be identified as arising from typical attractive or repulsive vdW interactions present among different moieties, and the red color represents the strong repulsion that appears in the center of the aromatic rings of the investigated rotaxane.

**Table 1:** Electron density *ρ(r)* (*e*·*a*_0_^−3^), Laplacian of electron density ∇^2^ *ρ(r)* (*e*·*a*_0_^−5^), electron kinetic energy density *G(r)* (hartree·*a*_0_^−3^), electron potential energy density *V(r)* (hartree·*a*_0_^−3^), and electron energy density *H(r)* (hartree·*a*_0_^−3^) for BCPs on selected bonds of the investigated [2]molecular shuttle calculated at B3LYP(D3)/cc‐pVTZ level from the crystallographic structure (CCDC number 2248267).^[^ ^21^ ^]^

BCPa)	Moietiesa)	Distance [Å]	*ρ*(*r*)	∇^2^ *ρ*(*r*)	*G*(*r*)	*V(r*)	*–G(r*)*/V(r*)	*H*(*r*)
N—H—C(sp^2^)	Bzi‐Stop	2.37	0.0135	0.0514	0.0104	−0.0079	1.3146	0.0025
HC—H—H—C(sp^3^)	Mac‐Stop	2.42	0.0047	0.0134	0.0028	−0.0021	1.2861	0.0006
HC—H—H—C(sp^3^)	Mac‐Stop	2.64	0.0030	0.0086	0.0017	−0.0012	1.3878	0.0005
HC—H—H—C(sp^3^)	Mac‐Stop	2.67	0.0028	0.0081	0.0016	−0.0011	1.3839	0.0004
HC—H—H—C(sp^3^)	Mac‐Stop	2.67	0.0023	0.0078	0.0015	−0.0010	1.5005	0.0005
HC—H—C(sp^2^)	Mac‐Stop	2.59	0.0095	0.0289	0.0059	−0.0047	1.2737	0.0013
HC—H—C(sp^2^)	Mac‐Stop	2.76	0.0068	0.0198	0.0040	−0.0030	1.3109	0.0009
HC—H—C(sp^2^)	Mac‐Stop	2.76	0.0068	0.0214	0.0043	−0.0032	1.3319	0.0011
HC—H—C(sp^2^)	Mac‐Stop	2.82	0.0062	0.0200	0.0040	−0.0030	1.3342	0.0010
HC—H—C(sp^2^)	Mac‐Stop	3.19	0.0031	0.0105	0.0020	−0.0014	1.4499	0.0006
HC—H—C(sp^2^)	Mac‐Stop	3.39	0.0024	0.0073	0.0014	−0.0009	1.4881	0.0005
HC—H—C(sp^2^)	Mac‐Stop	3.46	0.0022	0.0069	0.0013	−0.0009	1.4664	0.0004
HC—H—H—C(sp^2^)	Mac‐Stop	2.37	0.0058	0.0175	0.0036	−0.0028	1.2815	0.0008
O—C(sp^2^)	Mac‐Stop	3.32	0.0050	0.0166	0.0033	−0.0024	1.3767	0.0009
O—H—C(sp^2^)	Mac‐Stop	3.07	0.0029	0.0106	0.0021	−0.0015	1.3978	0.0006
O—H—C(sp^2^)	Mac‐Stop	2.56	0.0084	0.0303	0.0061	−0.0047	1.3094	0.0014
O—H—C(sp^2^)	Mac‐Stop	2.81	0.0047	0.0157	0.0032	−0.0025	1.2892	0.0007
O—H—C(sp^2^)	Mac‐Bipy	2.42	0.0101	0.0363	0.0073	−0.0056	1.3063	0.0017
O—H—C(sp^2^)	Mac‐Bipy	2.72	0.0053	0.0186	0.0037	−0.0028	1.3191	0.0009
O—C(sp^2^)	Mac‐Bipy	3.01	0.0082	0.0332	0.0065	−0.0046	1.3967	0.0018
O—C(sp^2^)	Mac‐Bipy	3.17	0.0059	0.0216	0.0042	−0.0030	1.3880	0.0012
O—C(sp^2^)	Mac‐Bipy	3.39	0.0045	0.0166	0.0033	−0.0024	1.3806	0.0009
O—H—C(sp^2^)	Mac‐Bipy	2.46	0.0096	0.0339	0.0069	−0.0054	1.2890	0.0016
O—H—C(sp^2^)	Mac‐Bipy	2.79	0.0047	0.0164	0.0033	−0.0025	1.3240	0.0008
HC—H—C(sp^2^)	Mac‐Bipy	2.81	0.0062	0.0226	0.0045	−0.0032	1.3742	0.0012
HC—H—C(sp^2^)	Mac‐Bipy	2.89	0.0056	0.0189	0.0037	−0.0027	1.3850	0.0010
O—N	Mac‐Bzi	2.94	0.0099	0.0387	0.0076	−0.0056	1.3627	0.0020
O—N	Mac‐Bzi	3.14	0.0066	0.0238	0.0047	−0.0035	1.3564	0.0012
O—N	Mac‐Bzi	3.23	0.0058	0.0202	0.0040	−0.0030	1.3357	0.0010
HC—H—N	Mac‐Bzi	2.73	0.0075	0.0297	0.0058	−0.0041	1.4055	0.0017
HC—H—N—H	Mac‐Bzi	2.75	0.0076	0.0321	0.0062	−0.0045	1.3987	0.0018
O—N—H	Mac‐Bzi	3.05	0.0077	0.0305	0.0059	−0.0042	1.4027	0.0017
O—H—N	Mac‐Bzi	2.06	0.0201	0.0778	0.0168	−0.0141	1.1877	0.0027
O—H—N	Mac‐Bzi	2.22	0.0141	0.0551	0.0113	−0.0089	1.2707	0.0024

a) The term “Mac” identifies the **24C8** macrocycle and “Stop” the stopper moieties.

A more punctual analysis of the NCI patterns may be derived by looking at the data collected in Table 1. The interface region between the rigid molecular thread and the **24C8** cycle includes numerous *ρ(r)* bond critical points (BCPs) of the type (+3, −1).^[^ ^27^ ^]^ In this table, we also report 1) the Laplacian of the density [$\nabla^{2}$ *ρ(r)*] as derived from the trace of the Hessian matrix of the *ρ(r)*; this allows an analysis of the curvatures of the *ρ(r)* at any estimated BCPs along the principal axes; 2) the values of the *G(r)* and *V(r*) to analyze the kinetic and potential energy densities; this for evaluating if interactions are controlled by a local reduction of the potential energy or by a local excess in the kinetic energy [*G(r) *> 0 and *V(r) *< 0, see Equation (3) and (4)]; 3) the total energy density *H(r*); see Equation (2), which is negative for interactions featuring a significant sharing of electrons, so to detect the covalent or partially covalent character of the interactions themselves. It is therefore evident from the QTAIM topological distribution that noncovalent forces act as a sort of glue connecting the cycle with the molecular thread in proximity to the symmetrical Bzi sites. As a matter of fact, at any estimated BCP, the *ρ(r)* is within 0.020 *e*·*a*_0_^−3^, the $\nabla^{2}$ *ρ(r)* and the *H*(*r*) are invariably positive, and the *–G(r)/V(r)* ratio results are greater than one. As a result, a large reservoir of (+3,−1) BCPs has emerged involving different functional groups: 1) 16 BPs^[^ ^28^ ^]^ are established by the **24C8** cycle (Mac) with the local stopper (Stop) moiety featuring interatomic distances between 2.37 and 3.46 Å; in particular, we are referring to HC—H—H—C(sp^3^), HC—H—C(sp^2^), HC—H—H—C(sp^2^), O—C(sp^2^), and O—H—C(sp^2^) weak vdW interactions of different nature. ii) 9 BPs [O—H—C(sp^2^), O—C(sp^2^), O—H—C(sp^2^) and HC—H—C(sp^2^)] are found to involve the Mac‐Bipy couple, and 8 BPs of the type O—N, HC—H—N, HC—H—N—H, O—N—H and O—H—N are found for the cycle and the Bzi recognition site; iii) a single inter‐moieties interaction also involve the N atom of the Bzi in the right side with an H—C(sp^2^) atom in the nearest stopper unit (see Figure 1c). Interestingly, we also observe that the strongest NCIs [*ρ(r) ≈ *0.014 and 0.020 *e*·*a*_0_^−3^] are those between the cycle and its preferential binding site, essentially reporting an H‐bond character and featuring the shorter distances observed (2.06 and 2.22 Å, see Table 1). The herein proposed analysis well highlights the existence of a rather complex supramolecular network finely modulating the mutual position of the **24C8** ether ring along the Bzi‐Bipy‐Bzi molecular thread. Moreover, the interaction patterns that have emerged clearly show at any CP a noncovalent character, the *H*(*r*) being positive, thus supporting the observed reversible shuttling detected by analyzing the ^1^H NMR spectra of such a synthetic system in DMF dilute solvent at room temperature.^[^ ^21^ ^]^ We mention here that we considered ‐ as a reference condition ‐ the deposited X‐ray configuration.

### 2.2 Theoretical Characterization of the [2]Rotaxane in the Presence of Zn(II)

**Figure 2:**
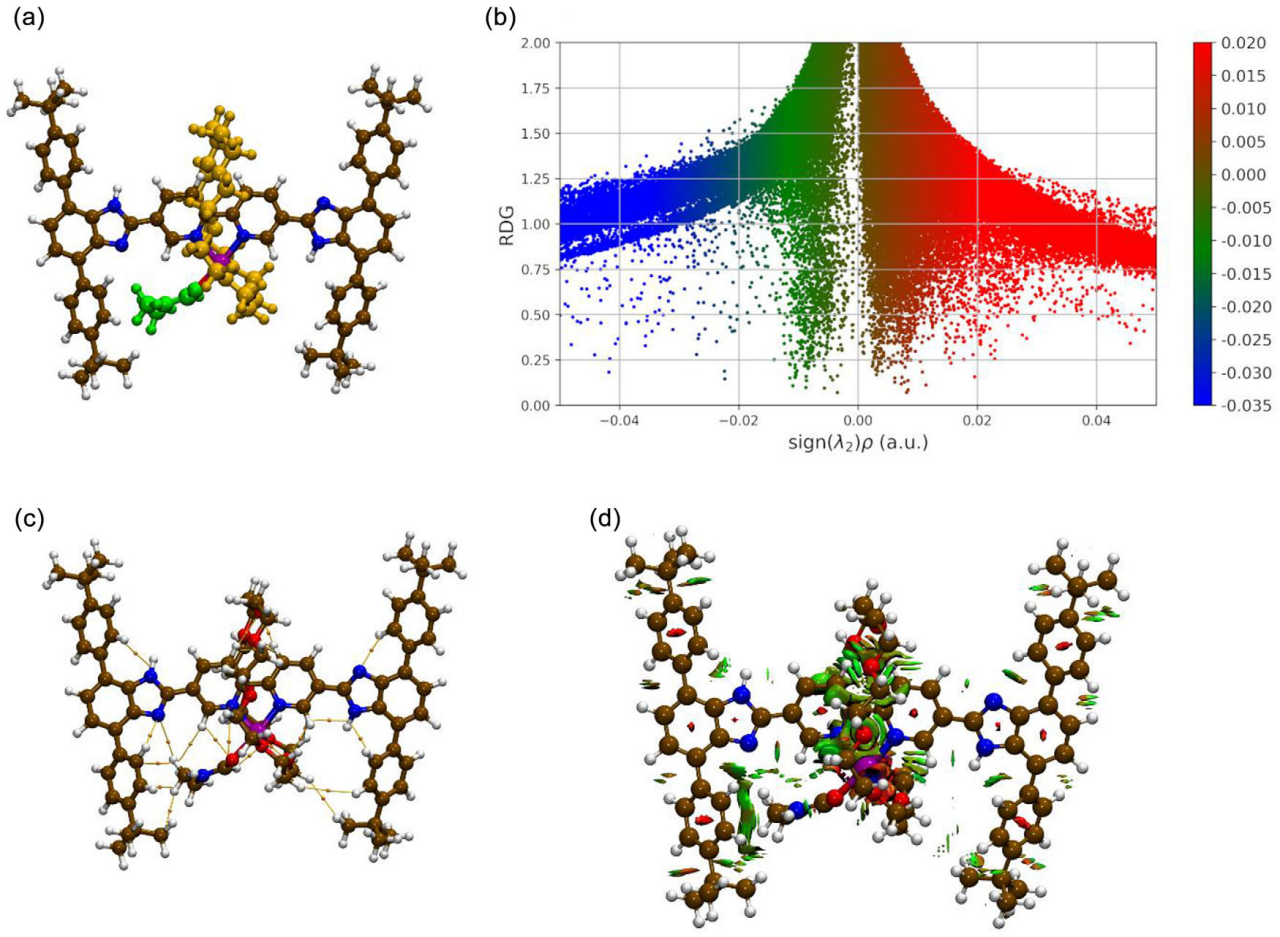
a) Molecular structure of the [2]molecular shuttle with Zn(II); the **24C8** ring is reported in orange while DMF is in green. b) The related 2D RDG plots in which the color bar represents the sign(*λ*_2_ *) × ρ(r)* in atomic units. c) Noncovalent BPs—at C‐PCM/B3LYP(D3)/cc‐pVTZ level—connecting the different moieties; the BPs are those analyzed in Table 2. d) 3D plot of NCIs characterizing supramolecular interaction patterns; blue refers to strong electrostatic attractions, green to vdW (either attractive or repulsive) interactions, and red to strong electrostatic repulsions.

EXSY spectrum reported evidences that in the presence of the [2]rotaxane, the addition of [Zn(NTf_2_)_2_] in a DMF dilute solution preserves the translocation of the macrocycle although with a slower shuttling rate of 2.2 s^−1^ than that previously observed only considering the free rotaxane (*i.e.,* 61.2 s^−1^).^[^ ^21^, ^29^ ^]^ This effect was attributed to the formation of an intermediate geometry in which the Zn(II) cation, after attachment to the nitrogen atoms of the Bipy chelate, can bind easily, in a reversible way, the crown ether during the shuttling translocation towards the two end points (i.e., Bzi units). In this respect, DFT simulations were carried out by Loeb and coworkers^[^ ^21^ ^]^ to support the presence of a ligand exchange mechanism involving DMF solvent molecules in coordination with the Zn(II) ion. Interestingly, the authors proposed a structure in which the Zn(II), at the center of an octahedral arrangement, coordinates three oxygen atoms of the crown ether, the Bipy chelate, and a molecule of DMF solvent. Starting from this result, we have 1) extended the accuracy of the DFT computations done previously, largely increasing the dimension of the atomic basis sets while maintaining the same functional and the continuum and polarizable dielectric algorithm (C‐PCM)^[^ ^30^, ^31^ ^]^ and 2) fully analyzed the optimized geometry by means of QTAIM‐based descriptors ‐ computed via Multiwfn program^[^ ^32^ ^]^ ‐ having the target of addressing the nature of the interactions characterizing such an intriguing midpoint between two translational isomers. In this respect, the optimized geometry in conjunction with the results carried out over the DFT‐derived converged wavefunction is reported in **Figure** **2** and **Table** **2**. For the sake of completeness, the DMF molecule bound to the metal ion is highlighted using green color atoms, and the QTAIM “observables” are the same as those in Figure 1b–d (i.e., free [2]rotaxane). We note that extending the accuracy of the computation in DMF, the optimized geometry presents a slight elongation (on average of 0.19 Å) of the Zn(II)–oxygen(Mac) contacts while the other distances remain substantially unaltered. The overall reservoir of NCIs that has emerged at C‐PCM/B3LYP(D3)/cc‐pVTZ level of theory is also displayed in Figure 2b together with the distribution of the BPs among the different interacting subunits. Looking at the data reported in this figure and collected in Table 2, it is evident the intriguing balance between multiple covalent and NCIs characterizing the investigated complex.

**Table 2:** Electron density *ρ(r)* (*e*·*a*_0_^−3^), Laplacian of electron density ∇^2^ *ρ(r)* (*e*·*a*_0_^−5^), electron kinetic energy density *G(r)* (hartree·*a*_0_^−3^), electron potential energy density *V(r)* (hartree·*a*_0_^−3^), and electron energy density *H(r)* (hartree·*a*_0_^−3^) for BCPs on selected bonds of the optimized [2]molecular shuttle with Zn(II) calculated at C‐PCM(DMF)/B3LYP(D3)/cc‐pVTZ level of theory.

BCP	Moietiesa)	Distance [Å]	*ρ*(*r*)	∇^2^ *ρ*(*r*)	*G*(*r*)	*V*(*r*)	*–G*(*r*)/*V*(*r*)	*H*(*r*)
Zn(II)—O=C (equat.)	Ion‐DMF	2.00	0.0703	0.3862	0.1011	−0.1057	0.9567	−0.0046
Zn(II)—N (equat.)	Ion‐Bipy	2.07	0.0751	0.3254	0.0935	−0.1056	0.8854	−0.0121
Zn(II)—N (equat.)	Ion‐Bipy	2.09	0.0720	0.3106	0.0882	−0.0987	0.8933	−0.0105
Zn(II)—O (equat.)	Ion‐Mac	2.20	0.0465	0.2084	0.0529	−0.0537	0.9847	−0.0008
Zn(II)—O (axial)	Ion‐Mac	2.26	0.0448	0.2127	0.0529	−0.0534	0.9904	−0.0005
Zn(II)—O (axial)	Ion‐Mac	2.34	0.0293	0.1093	0.0277	−0.0280	0.9876	−0.0003
H_2_C—H—N	DMF‐Bzi	2.72	0.0071	0.0201	0.0046	−0.0041	1.1186	0.0005
H_2_C—H—H—C(sp^2^)	DMF‐Bipy	2.49	0.0043	0.0172	0.0033	−0.0022	1.4771	0.0011
C=O—H—C(sp^2^)	DMF‐Bipy	2.53	0.0095	0.0341	0.0077	−0.0069	1.1138	0.0008
H_2_C—H—H—CH_2_	DMF‐Stop	2.44	0.0047	0.0174	0.0034	−0.0024	1.4170	0.0010
H_2_C—H—C(sp^2^)	DMF‐Stop	2.88	0.0059	0.0190	0.0037	−0.0027	1.3769	0.0010
H_2_C—H—C(sp^2^)	DMF‐Stop	2.95	0.0057	0.0185	0.0036	−0.0027	1.3726	0.0010
O=C—H—O	DMF‐Mac	2.45	0.0116	0.0393	0.0092	−0.0086	1.0722	0.0006
O=C—H—C(sp^3^)	DMF‐Mac	2.79	0.0035	0.0138	0.0024	−0.0014	1.7467	0.0010
O—H—C(sp^2^)	Mac‐Bipy	2.03	0.0229	0.0723	0.0174	−0.0168	1.0381	0.0006
O—H—C(sp^2^)	Mac‐Bipy	2.36	0.0132	0.0419	0.0102	−0.0099	1.0304	0.0003
O—H—C(sp^2^)	Mac‐Bipy	2.37	0.0123	0.0336	0.0086	−0.0085	1.0129	0.0001
O—H—C(sp^2^)	Mac‐Bipy	2.52	0.0103	0.0432	0.0089	−0.0071	1.2631	0.0019
O—H—C(sp^2^)	Mac‐Bipy	2.53	0.0099	0.0392	0.0084	−0.0070	1.2038	0.0014
O—H—C(sp^2^)	Mac‐Bipy	2.78	0.0056	0.0235	0.0048	−0.0038	1.2687	0.0010
C(sp^3^)—H—N	Mac‐Bipy	2.71	0.0089	0.0288	0.0062	−0.0052	1.1906	0.0010
C(sp^3^)—H—N	Mac‐Bipy	2.77	0.0068	0.0224	0.0047	−0.0039	1.2181	0.0008
C(sp^3^)—H—C—N	Mac‐Bipy	2.95	0.0046	0.0190	0.0034	−0.0020	1.6603	0.0013
H—C(sp^3^)—H—C(sp^2^)	Mac‐Bipy	2.99	0.0055	0.0162	0.0032	−0.0024	1.3332	0.0008
O—C(sp^2^)	Mac‐Bipy	3.01	0.0093	0.0319	0.0068	−0.0057	1.1957	0.0011
O—C—N	Mac‐Bipy	3.03	0.0088	0.0299	0.0064	−0.0053	1.2033	0.0011
O—C—H	Mac‐Bipy	3.16	0.0069	0.0291	0.0058	−0.0042	1.3615	0.0015
C(sp^3^)—H—C	Mac‐Bipy	3.74	0.0029	0.0099	0.0019	−0.0013	1.4718	0.0006
C(sp^3^)—H—H—C(sp^3^)	Mac‐Stop	2.77	0.0023	0.0087	0.0015	−0.0009	1.7151	0.0006
H—C(sp^3^)—H—C(sp^2^)	Mac‐Stop	3.13	0.0009	0.0035	0.0006	−0.0003	2.1660	0.0003
H—N—H—C(sp^2^)	Bzi‐Stop	2.65	0.0088	0.0333	0.0068	−0.0053	1.2858	0.0015
N—H—C(sp^2^)	Bzi‐Stop	2.69	0.0109	0.0375	0.0081	−0.0069	1.1843	0.0013
N—C(sp^2^)	Bzi‐Stop	2.73	0.0076	0.0345	0.0065	−0.0045	1.4629	0.0021
H—N—C(sp^2^)—H	Bzi‐Stop	3.36	0.0089	0.0388	0.0075	−0.0052	1.4264	0.0022
H—N—H—C	Bzi‐Bipy	2.63	0.0098	0.0459	0.0091	−0.0067	1.3597	0.0024

a) The term “Mac” identifies the **24C8** macrocycle and “Stop” the stopper moieties.

At first, it is worth noting that the *H*(*r*) in Table 2 reports negative values only for (+3, −1) BCPs connecting the Zn(II) cation with the rotaxane and the introduced DMF solvent molecule. However, these data reveal that the most prominent interactions showing a covalent character are those connecting the Zn(II) species with the N atoms of the Bipy unit [*H*(*r*) = −0.0121 and −0.0105 hartree·*a*_0_^−3^] and with the carbonyl O atom of the coordinated DMF molecule [*H*(*r*) = −0.0046 hartree·*a*_0_^−3^] in the equatorial part of the predicted octahedral complex; the associated *ρ(r)* are estimated—at the BCPs—at almost 0.07 *e*·*a*_0_^−3^. The remaining three interaction patterns at 2.20 (equatorial), 2.26 and 2.33 Å (axial) with the O atoms of the ether ring suggest evidence for covalence, albeit of a lesser extent [*H*(*r*) takes on negative values centered at about −0.0005 hartree·*a*_0_^−3^] when compared with contacts at shorter distances and involving both Bipy and DMF. The derived *ρ(r)* are estimated in the 0.046–0.029 *e*·*a*_0_^−3^ range; the ∇^2^ *ρ(r)* values result in positive, and the *–G(r)/V(r)* ratio is slower than one. The trend observed via QTAIM analysis provides a self‐consistent theoretical picture supporting the prediction of a ligand exchange mechanism in such a [2]rotaxane following the addition of Zn(II) cations. In fact, data collected in Table 2 clearly evidence that although the **24C8** macrocycle unit participates in the coordination with the metal ion, it substantially forms much weaker bonds. The overall geometry of such a transient complex is further stabilized by a significant amount of NCIs involving different functional groups among different moieties (see the BPs reported in Figure 2c and further specified in Table 2). In this respect, the DMF molecule was also found to interact with Bzi, Bipy, the Stopper, and the macrocycle; in particular, our computations reveal the formation of an inter‐moieties H‐bonding O=C—H—O contact (with a distance of 2.45 Å) between the DMF‐Mac couple, see Table 2. In any case, the majority of supramolecular contacts involve the **24C8** and Bipy unit. Moreover, contrary to what was observed for the free [2]rotaxane (see Figure 1 and data collected in Table 1), no H‐bonds are observed between the **24C8** cycle and the Bzi units.

**Figure 3:**
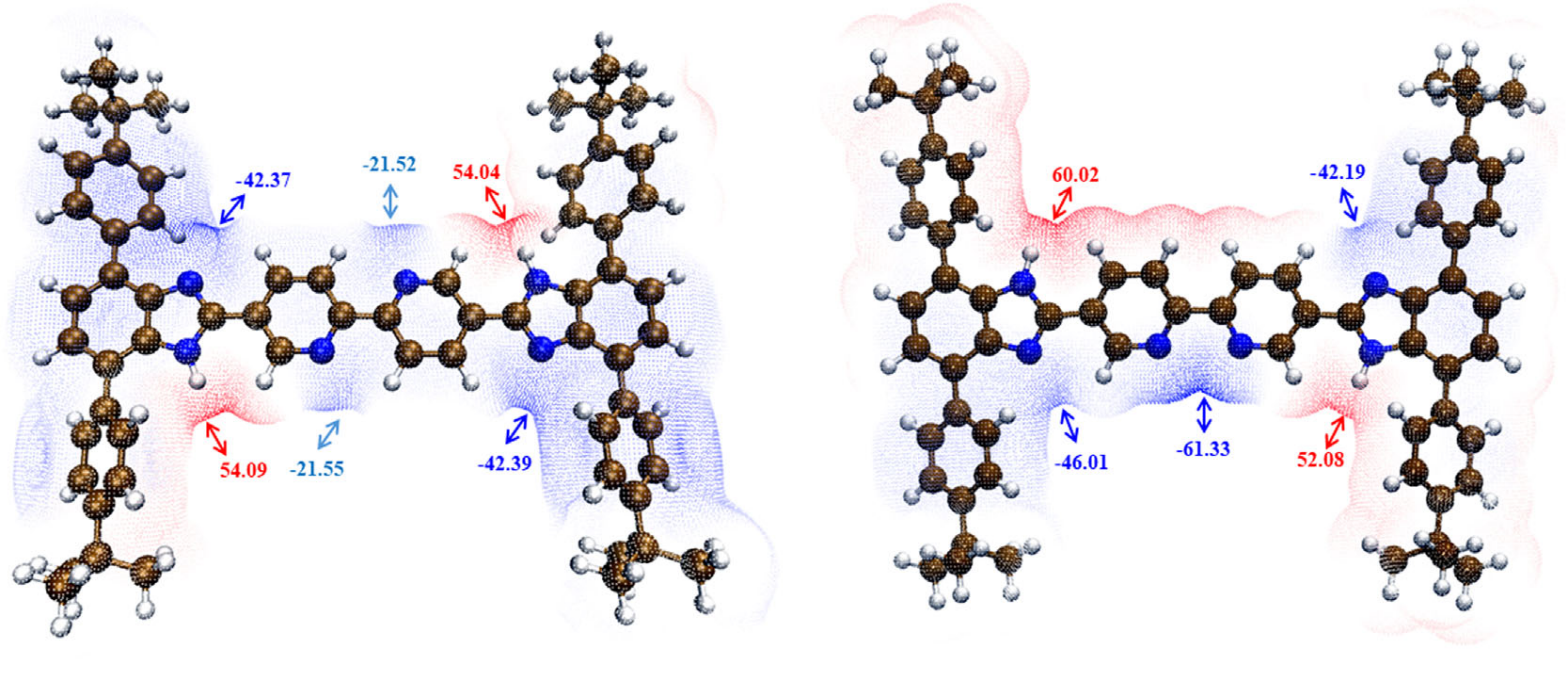
Electrostatic potential (ESP, in kcal mol^−1^) colored electron density isosurface (isodensity value of 0.03 a.u.) for the Stop‐[Bzi‐Bipy‐Bzi]‐Stop molecular thread in two different conformations encircled by a DMF dielectric environment at C‐PCM/B3LYP(D3)/VTZ level of theory. The blue and red labels represent the local minima and maxima points of the ESP surface, respectively.

The electrostatic potential energy (ESP) surfaces for the Stop‐[Bzi‐Bipy‐Bzi]‐Stop molecular thread in two different conformations are estimated with the Multiwfn program,^[^ ^32^ ^]^ and the results are displayed in **Figure** **3**. These surfaces represent the charge distribution of such a rigid thread, indicating its local properties and how it interacts with other species. More in detail, electrophilic‐based reactivity (or complexation) is actually represented by blue color, indicating negative regions of the molecule that experience stronger repulsion and are located on electronegative atoms typically rich in electrons. On the other hand, positive values (red color) suggest—on average—a lack of electrons within local regions, thus being prone to nucleophilic reactivity. This plot shows that *cis/trans* isomerization of the Bipy unit alters the ESP value of such a thread; we note that in *cis* conformation the Bipy is capable of binding metals as a chelating ligand forming a 5‐membered chelate ring. The ESP value around nitrogen atoms undergoes a significant variation from almost −21.5 to −61.33 kcal mol^−1^ when passing from *trans* to *cis* isomers of neutral Bipy (see Figure 3). This trend results in line with the observed cation complexation following the addition of Zn(II) and Pt(II) in the presence of the free [2]rotaxane in DMF.^[^ ^21^ ^]^ Moreover, the ESP surfaces reflect the peculiar electrophilic versus nucleophilic character of the imposed functional recognition sites in the presence of the **24C8** ring. In fact, the estimated trend unequivocally reveals that, under exercise conditions, the two N—H functional groups within the Bzi moieties can be attractive for a nucleophilic‐driven complexation by the mechanically interlocked **24C8** macrocycle.

The small asymmetries observed in the *trans* conformation (Figure 3, left panel) are ascribed to the fact that we do not impose any symmetrical constraint in the molecular structure of the Stop‐[Bzi‐Bipy‐Bzi]‐Stop thread during optimization procedures carried out at the C‐PCM/B3LYP(D3)/VTZ level of theory. Our investigation also highlights that the two Bzi recognition sites may also express a different affinity with respect to an electrostatic‐driven complexation when an asymmetrical *cis* conformation is concerned. As a matter of fact, the N—H group of the Bzi station when located on the same side of the Bipy chelating region shows a slight attenuation of the positive ESP value if compared with the same chemical group when localized on the opposite side (52.08 versus 60.02 kcal mol^−1^, see Figure 3).^[^ ^33^, ^34^ ^]^

**Figure 4:**
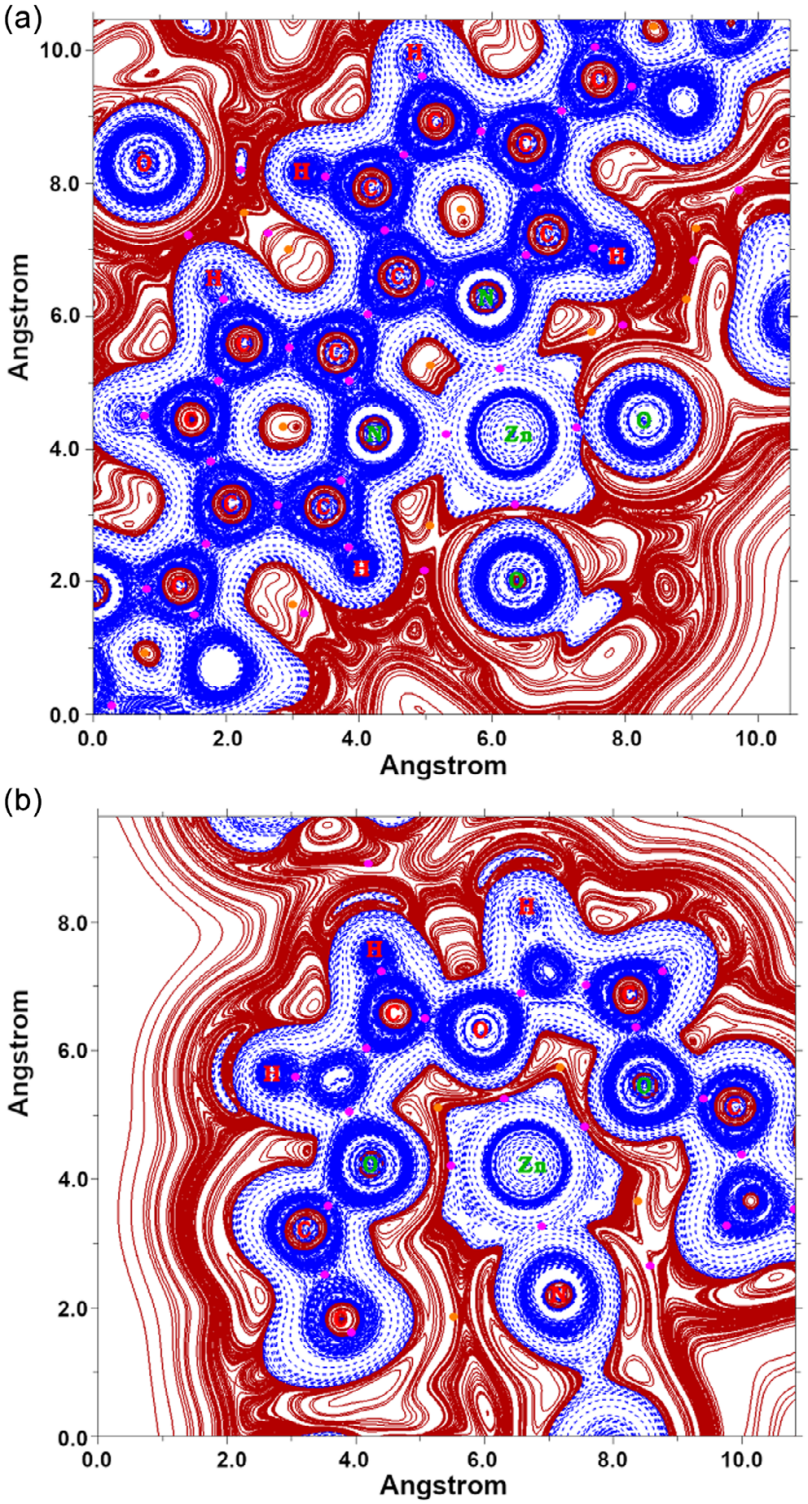
(a) 2D plot of the C‐PCM‐B3LYP(D3)/cc‐pVTZ *H(r) = G(r) + V(r)* in the σ plane defined by the position of N(Bipy)‐Zn^2+^‐O(DMF) atoms in the optimized geometry. The O(DMF) is located approximately at the coordinate point (*X* = 8.1; *Y* = 4.2). Solid/brown and dashed/blue lines correspond, respectively, to positive and negative values of the *H(r)* function. The dots in magenta indicate the CPs (+3, −1) along predefined BPs (see also Figure 2 and data in Table 2). (b) 2D plot of the *H(r)* function over a plane defined by O(Mac)‐Zn^2+^‐O(Mac) atoms. Oxygen atoms in the equatorial and axial sites are reported in green. Please note that, for the sake of clearness, Figure S5 reported as additional material explicitly shows the *H(r)* isovalue associated on a subset of imposed contour lines.

At last, we further investigated the chemical interactions connecting the Zinc(II) cation with the encircling environment analyzing the topology of the *H*(*r*) function as extracted from the converged electronic wavefunction at the C‐PCM/B3LYP level of computation. To this end, in **Figure** **4**, we report the plot of the *H(r)* function over either the *σ*[N(Bipy)‐Zn^2+^‐O(DMF)] or *σ*[O(Mac)‐Zn^2+^‐O(Mac)] plane, respectively; in this respect, typical *H(r)* contour lines belonging to the pattern *±k* × 10^*n*^ (*k = *0, 1, 2, 4, 8; *n* = −5 to 6) are used.^[^ ^35^ ^]^ The first plot while reporting the *H(r)* function over the equatorial plane of the octahedral metal complex unequivocally allows an analytical discretization of the bonding motifs characterizing the first solvation shell: 1) as it can be clearly observed in Figure 4a, the BCPs along the Zn^2+^—N(Bipy) BPs are located in a continuous region of negative values of *H(r)*, −0.0121 and ‐0.0105 hartree·*a*_0_^−3^ (see Table 2), thus suggesting the presence of covalent interactions between the cation and the N atoms of the Bipy chelating species; 2) regarding the Zn^2+^—O=C(DMF), the negative value of the *H(r) =* −0.0046 hartree·*a*_0_^−3^ at the BCP coupled with the trend displayed in the figure let us plausibly suppose the existence of a rather confined covalent‐based interaction encircled by regions of positive values [i.e., *H* ^*+*^ *(r)*]; 3) a different interaction pattern may be observed along the equatorial site connecting the ether O atom of the ring with the metallic ion. In fact, considering the Zn^2+^—O(Mac) interacting couple even if the value of *H(r)* is negative at the BCP (−0.0008 hartree·*a*_0_^−3^ as reported in Table 2), there is no evidence of an underlying covalent contact. The value of the *H(r)* at the BCP is less negative by more than an order of magnitude when compared to that between Zn^2+^—N(Bzy) and Zn^2+^—O=C(DMF). In this respect, the plot of the *H(r)* shows that, at the BCP, Zn^2+^ and O(Mac) overlap only part of their *H* ^*+*^ *(r)* regions, while their *H* ^*−*^ *(r)* domains remain closed and well separated by a region of positive values of *H(r)*. As already observed over similar systems containing Ag^+^ and Pd^2+^, this is a representative example of rather weak noncovalent contacts of variable nature.^[^ ^36^, ^37^ ^]^

The same behavior is observed along the two axial sites occupied by ether O atoms of the **24C8** ring (see Figure 4b); the values of the *H(r)* function at the BCPs are estimated at −0.0005 and −0.0003 hartree·*a*_0_^−3^ and, most importantly, the interatomic species do not overlap continuous regions of negative values of the *H(r)*. This aspect is crucial to explain, from a first‐principles approach based on electronic density estimated at a quantum mechanical level, the weak nature of the Zn^2+^—24C8 contacts which then have the real effect of enabling the shuttling process (although it is slowed down by the presence of the metal cation) observed in a DMF dilute solution at room temperature. Bond order (BO) analysis reported in the Supporting Information further confirms this picture. On the other hand, coordination of a PtCl_2_ moiety to the Bipy chelating unit in a square planar geometry generated an insurmountable steric barrier to shuttling motion.^[^ ^21^ ^]^ Interestingly, no evidence of macrocycle shuttling translocation between the Bzi recognition sites was observed even at increased temperatures as witnessed by EXSY (EXchange SpectroscopY) NMR experiments. Finally, as a concluding remark, it is worth mentioning that 1) conceptual DFT (CDF) descriptors^[^ ^38^ ^]^ support the nucleophilicity character in proximity to the unsaturated N atoms within the Bipy chemical unit already appreciated by plotting the ESP (see Figure 3),^[^ ^39^, ^40^ ^]^ and 2) further computations carried out by applying a parameter‐free hybrid functional substantially provide the same interaction scenario estimated with the B3LYP functional both in terms of structural parameters and QTAIM indices.^[^ ^41^, ^42^ ^]^

## 3 Conclusion

In this contribution we report direct evidence—by means of converged electronic DFT wavefunctions in conjunction with QTAIM descriptors—of the intrinsic weakness of the inter‐moieties contacts characterizing a plausible octahedral intermediate compound at the midpoint along a shuttling process involving a **24C8** macrocycle over a symmetric Bzi‐Bipy‐Bzi molecular thread. As a result, the proposed computational scenario strongly supports the existence of a fascinating ligand exchange mechanism allowing the translocation of the ring in DMF also in the presence of a chelating species for Zn(II) cations as well as the Bipy subunit. In addition, we also fully characterized the NCIs modulating the conformational shaping in such a [2]rotaxane taking, as reference conditions, the only available X‐ray structure reported in literature. The overall picture that has emerged suggests that intensive numerical simulations allow accurate analyses of structural and electronic properties in complex molecular systems for nanotechnological applications. In this respect, further theoretical investigations coupling DFT electronic degrees of freedom and classical molecular dynamics techniques are necessary for a complete understanding of the intricate balance between conformational shaping and dynamical responses in the investigated H‐shaped [2]rotaxane. In this general context, an interplay of theoretical modeling and experiments might open up new strategies for the design of ecosustainable stimuli‐responsive molecular devices and machines with improved efficiencies and functionalities.

## 4 Computational Section

Supramolecular interaction patterns modulating the conformational shaping in the investigated [2]rotaxane molecular shuttle with a central Bipy rigid core were addressed by using a computational procedure recently applied over a pH‐ and metal‐actuated molecular shuttle.^[^ ^36^ ^]^ In this specific case, we considered as reference condition the crystallographic structure available in literature unequivocally showing the **24C8** macrocycle encircling one of two Bzi stations (CCDC number—2248267).^[^ ^21^ ^]^ The derived DFT‐based indicators, at B3LYP(D3)^[^ ^43^, ^44^, ^45^, ^46^, ^47^ ^]^/cc‐pVTZ^[^ ^48^ ^]^ level of theory, included the electron density *ρ*(*r*), the electronic energy density *H*(*r*), and its kinetic and potential components *G*(*r*) and *V*(*r*), respectively, and the reduced density gradient (RDG) *s*(*r*). The ρ(*r*) is defined by the equation
(1)

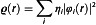

where $\eta_{i}$ is the occupation number of the natural orbital $\varphi_{i}$, in turn expanded as a linear combination of the atomic basis functions.^[^ ^23^ ^]^ The *H(r)* is the sum of the kinetic energy density *G(r)* and the potential energy density *V(r)*

(2) H(r)=G(r)+V(r)

The presently used definition^[^ ^23^, ^24^, ^25^, ^49^ ^]^ of the *G(r)* was given by the equation
(3)

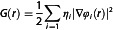

with the sum running over all the occupied natural orbitals $\varphi_{i}$ of occupation numbers $\eta_{i}$. The potential energy density *V(r)* was evaluated^[^ ^23^, ^24^, ^25^ ^]^ from the local form of the virial theorem

(4) $$V\left(r\right)=\frac{1}{4}\nabla^{2}\rho \left(r\right)-2G\left(r\right)$$

The RDG—coming from the density and its first derivative—was defined by the equation^[^ ^50^ ^]^

(5) $$s\left(r\right)=\frac{\left|\nabla \rho \left(r\right)\right|}{2{\left(3\pi^{2}\right)}^{\frac{1}{3}}\times \rho {\left(r\right)}^{\frac{4}{3}}}$$

Low‐value of *s(r)* isosurfaces (typically 0.3–0.6) appeared among atoms undergoing any type of interaction, the noncovalent interactions (NCIs) emerging, in particular, by considering the spatial regions of low *ρ(r)* (typically at around 0.05 *e*·*a*_0_^−3^). The low‐*s(r)/*low*‐ρ(r)* isosurfaces were, in turn, mapped in terms of the sign(*λ*_2_ *) × ρ(r), λ*_2_ being the second eigenvalue (*λ*_1_ *< λ*_2_ *< λ*_3_) of the Hessian matrix of *ρ(r).* In essence, the sign of *λ*_2_ was used to distinguish between attractive (*λ*_2_ < 0) and repulsive (*λ*_2_ > 0) interactions, and the value of *ρ(r)* was exploited to rank the corresponding strength. Thus, the NCIs were conveniently visualized in a 2D plane by plotting the RDG versus the colored sign(*λ*_2_ *) × ρ(r)* and in 3D space by plotting a chosen low‐value isosurface of the *s(r)*, colored by the sign(*λ*_2_ *) × ρ(r)* (standard used colors range from blue to red for attractive to repulsive interactions, respectively). The analysis was done with the Multiwfn program (version 3.8.dev)^[^ ^32^ ^]^ and DFT computations with the Gaussian 16 code (Rev. C01).^[^ ^51^ ^]^ The 2D and 3D plots of the *s(r)* versus sign(*λ*_2_ *) × ρ(r)* were realized with Atomistica.online^[^ ^52^ ^]^ and the Visual Molecular Dynamics program^[^ ^53^ ^]^ setting sign(*λ*_2_ *) × ρ(r)* between −0.050 *e*·*a*_0_^−3^ (blue) and 0.010 *e*·*a*_0_^−3^ (red). We finally underlined that the reduced computational cost recommended DFT calculations to investigate NCIs in large and complex molecular systems like the present molecular rotaxane.

We also focused our attention on the proposed transient complex species in which the Bipy central core chelates Zn(II) cations in an octahedral arrangement together with a solvent molecule and ether oxygen atoms forming the **24C8** cycle.^[^ ^54^ ^]^ In this respect, the *H(r)* function was plotted over two different planes defining axial and equatorial positions of the ligands. Such a function partitioned the atomic space into inner regions featuring negative values, indicated as *H* ^*−*^ *(r)*, and outer regions of positive values, indicated as *H* ^*+*^ *(r)*. When two atoms form a chemical bond, these regions combined in ways that signal the nature of the interaction.^[^ ^35^, ^36^ ^]^ This graphical representation provided a simple and intuitive picture to rationalize the differences between covalent and noncovalent coordination sites for the Zn(II) cation in the optimized structure of the investigated octahedral intermediate.

## Conflict of Interest

The authors declare no conflict of interest.

## Supporting information

Supplementary Material
