# Supplementary material for: QTAIM Analysis of a [2]Rotaxane Molecular Shuttle with a 2,2′‐Bipyridyl Rigid Core
Source: Chemphyschem. 2025 Apr 21;26(12):e202500074. doi: 10.1002/cphc.202500074 (PMC12188175; doi:10.1002/cphc.202500074)
Supplement: Supplementary file 1 — Supplementary Material [file CPHC-26-e202500074-s001.pdf]

QTAIM analysis of a [2]rotaxane molecular shuttle with a 2,2'-bipyridyl rigid core

Costantino Zazza,*[a] Nico Sanna,[a,b] Stefano Borocci[a,c] and Felice Grandinetti[a,c]

[a] Dr. Costantino Zazza, Prof. Nico Sanna, Prof. Stefano Borocci, and Prof. Felice Grandinetti
Department for Innovation in Biological, Agro-food and Forest systems, Università della Tuscia (DIBAF), L.go dell'Università, s.n.c., 01100 Viterbo, Italy.
E-mail: costantino.zazza@unitus.it

[b] Prof. Nico Sanna
CNR-ISTP (Istituto per la Scienza e Tecnologia dei Plasmi), Via G. Amendola 122/D, 70126 Bari, Italy.

[c] Prof. Stefano Borocci and Prof. Felice Grandinetti
Istituto per i Sistemi Biologici del CNR (ISB), Sede di Roma - Meccanismi di Reazione c/o Dipartimento di Chimica, Sapienza Università di Roma, P.le A. Moro 5, Rome (Italy).

Electronic Supporting Information

Table S1 showing the electronic transitions at TD-B3LYP(D3)/cc-pVTZ for the investigated [2]rotaxane molecular shuttle with a 2,2'-bipyridyl rigid core (CCDC number 2248267); note that we only show excitation with oscillator strength > 0.1.

No,	Wavelength (nm)	Osc, Strength	Symmetry	Major contribs
1	397.8	0.355	Singlet-A	HOMO->LUMO (96%)
2	360.2	0.641	Singlet-A	H-1->LUMO (95%)
3	349.1	0.277	Singlet-A	H-2->LUMO (95%)
4	334.4	0.312	Singlet-A	HOMO->L+1 (69%), HOMO->L+2 (24%)
6	317.2	0.090	Singlet-A	HOMO->L+1 (22%), HOMO->L+2 (64%)
7	316.8	0.405	Singlet-A	H-3->LUMO (70%)
10	303.9	0.468	Singlet-A	H-2->L+2 (10%), H-1->L+1 (73%)
11	299.6	0.201	Singlet-A	H-2->L+1 (57%), H-1->L+1 (11%), H-1->L+2 (27%)
12	289.9	0.132	Singlet-A	H-2->L+1 (12%), H-1->L+2 (28%), H-1->L+3 (44%)
				HOMO is 341

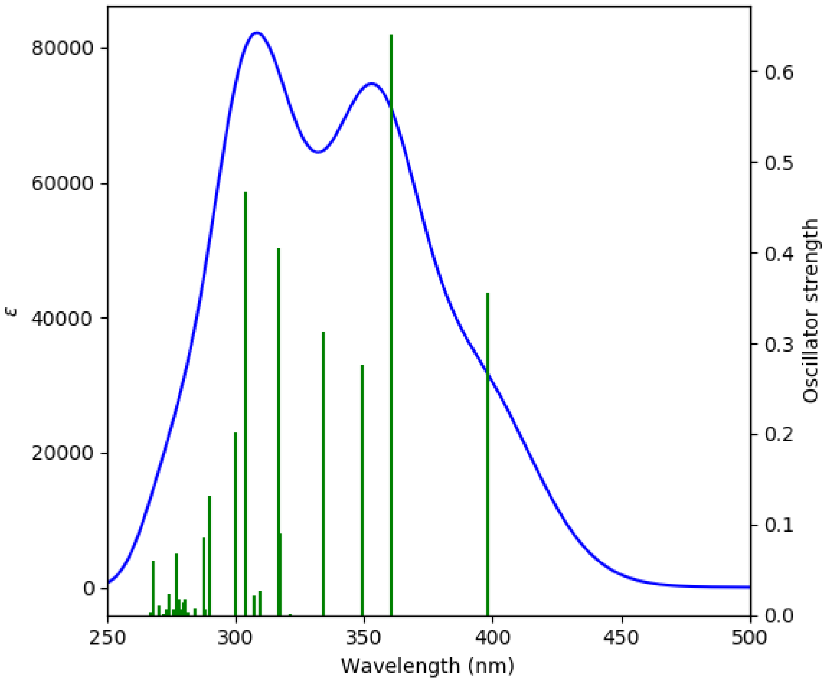

Figure S1. UV/Vis absorption spectra (please see also data collected in Table S1) of the investigated [2]rotaxane. The molar absorption coefficient (Ɛ) is reported in $M^{-1}cm^{-1}$. Oscillator strengths (in a.u.) are also reported as green histograms.

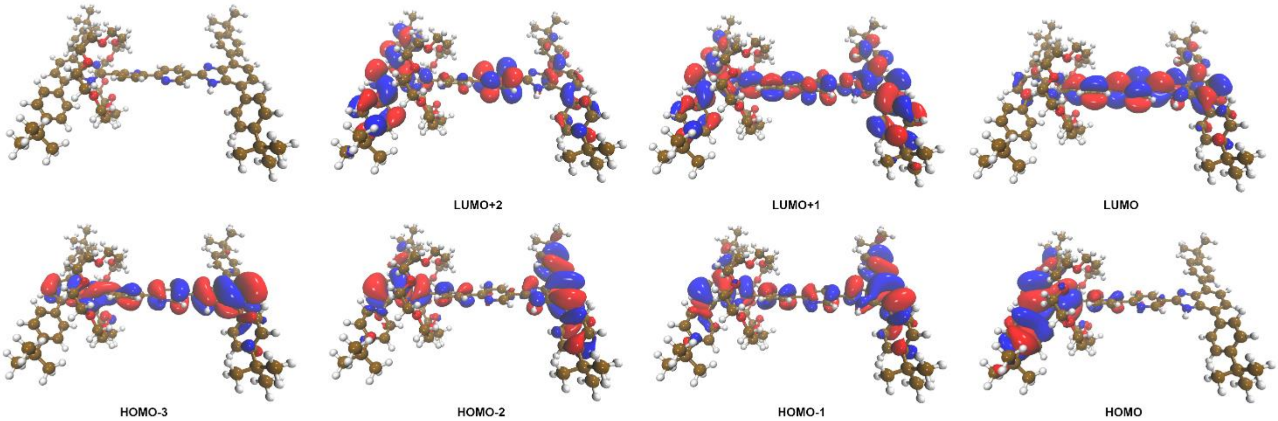

Figure S2 Frontier Molecular Orbitals (FMOs) of the investigated [2]molecular shuttle at B3LYP(D3)/cc-pVTZ level of theory. The blue colour reflects the positive (+) part of the corresponding eigenvector and an isodensity value of 0.015 $e{\cdot}a_0^3$ is used.

Optimized geometry - at C-PCM(DMF)/B3LYP(D3)/cc-pVTZ level of theory - of the investigated [2]rotaxane in the presence of Zn(II).

1(Zn) --> Charge: 20.000000 x,y,z(Bohr): 0.150434 3.508477 -0.142946
2(N) --> Charge: 7.000000 x,y,z(Bohr): 2.709779 0.553644 -0.276533
3(N) --> Charge: 7.000000 x,y,z(Bohr): -2.292854 0.416758 -0.149583
4(N) --> Charge: 7.000000 x,y,z(Bohr): -10.178053 1.024967 -0.906867
5(N) --> Charge: 7.000000 x,y,z(Bohr): -10.818958 -3.115976 -0.264843
6(H) --> Charge: 1.000000 x,y,z(Bohr): -10.438118 -4.933622 0.199053
7(N) --> Charge: 7.000000 x,y,z(Bohr): 10.609909 1.578278 0.249129
8(H) --> Charge: 1.000000 x,y,z(Bohr): 9.667053 3.199115 0.629777
9(N) --> Charge: 7.000000 x,y,z(Bohr): 11.361645 -2.573036 -0.180604
10(C) --> Charge: 6.000000 x,y,z(Bohr): 1.664487 -1.774518 -0.148043
11(C) --> Charge: 6.000000 x,y,z(Bohr): 3.189455 -3.925609 0.079976
12(H) --> Charge: 1.000000 x,y,z(Bohr): 2.341790 -5.790597 0.249645
13(C) --> Charge: 6.000000 x,y,z(Bohr): 5.803858 -3.642694 0.112952

14(H) --> Charge: 1.000000 x,y,z(Bohr):   7.036012 -5.284648   0.292897
15(C) --> Charge: 6.000000 x,y,z(Bohr):   6.876181 -1.220820 -0.072928
16(C) --> Charge: 6.000000 x,y,z(Bohr):   5.213998   0.829258 -0.272746
17(H) --> Charge: 1.000000 x,y,z(Bohr):   5.864972   2.777232 -0.407672
18(C) --> Charge: 6.000000 x,y,z(Bohr):   9.602203 -0.802312 -0.016561
19(C) --> Charge: 6.000000 x,y,z(Bohr): 13.201247   1.338391   0.209381
20(C) --> Charge: 6.000000 x,y,z(Bohr): 13.647158 -1.291120 -0.055046
21(C) --> Charge: 6.000000 x,y,z(Bohr): 16.161306 -2.218087 -0.128292
22(C) --> Charge: 6.000000 x,y,z(Bohr): 18.069553 -0.390770   0.115747
23(H) --> Charge: 1.000000 x,y,z(Bohr): 20.036500 -1.013604   0.122792
24(C) --> Charge: 6.000000 x,y,z(Bohr): 17.569677   2.211292   0.392620
25(H) --> Charge: 1.000000 x,y,z(Bohr): 19.157017   3.518737   0.563323
26(C) --> Charge: 6.000000 x,y,z(Bohr): 15.106055   3.177962   0.427795
27(C) --> Charge: 6.000000 x,y,z(Bohr): 14.458546   5.884710   0.737961
28(C) --> Charge: 6.000000 x,y,z(Bohr): 15.620328   7.395905   2.581800
29(H) --> Charge: 1.000000 x,y,z(Bohr): 17.090353   6.583377   3.781823
30(C) --> Charge: 6.000000 x,y,z(Bohr): 14.862398   9.899335   2.971504
31(H) --> Charge: 1.000000 x,y,z(Bohr): 15.800751 10.970535   4.458492
32(C) --> Charge: 6.000000 x,y,z(Bohr): 12.919336 11.006520   1.540980
33(C) --> Charge: 6.000000 x,y,z(Bohr): 11.802715   9.497816 -0.344061
34(H) --> Charge: 1.000000 x,y,z(Bohr): 10.298594 10.269246 -1.526626
35(C) --> Charge: 6.000000 x,y,z(Bohr): 12.552554   7.007429 -0.747245
36(H) --> Charge: 1.000000 x,y,z(Bohr): 11.656685   5.926777 -2.261020
37(C) --> Charge: 6.000000 x,y,z(Bohr): 11.941595 13.706335   1.985652
38(C) --> Charge: 6.000000 x,y,z(Bohr):   9.064981 13.595177   2.494297
39(H) --> Charge: 1.000000 x,y,z(Bohr):   8.656190 12.457941   4.190826
40(H) --> Charge: 1.000000 x,y,z(Bohr):   8.026671 12.755254   0.896781
41(H) --> Charge: 1.000000 x,y,z(Bohr):   8.312334 15.513339   2.801316
42(C) --> Charge: 6.000000 x,y,z(Bohr): 13.227138 14.991393   4.255803
43(H) --> Charge: 1.000000 x,y,z(Bohr): 12.493414 16.926930   4.470960
44(H) --> Charge: 1.000000 x,y,z(Bohr): 15.289090 15.123624   4.002073
45(H) --> Charge: 1.000000 x,y,z(Bohr): 12.852501 13.976536   6.033812
46(C) --> Charge: 6.000000 x,y,z(Bohr): 12.443709 15.327045 -0.393239
47(H) --> Charge: 1.000000 x,y,z(Bohr): 11.485016 14.560565 -2.072414
48(H) --> Charge: 1.000000 x,y,z(Bohr): 14.482784 15.424409 -0.805093
49(H) --> Charge: 1.000000 x,y,z(Bohr): 11.748037 17.268758 -0.099232
50(C) --> Charge: 6.000000 x,y,z(Bohr): 16.758360 -4.936371 -0.423240
51(C) --> Charge: 6.000000 x,y,z(Bohr): 18.894551 -5.724756 -1.801972
52(H) --> Charge: 1.000000 x,y,z(Bohr): 20.091481 -4.314337 -2.718946
53(C) --> Charge: 6.000000 x,y,z(Bohr): 19.481289 -8.278861 -2.058159
54(H) --> Charge: 1.000000 x,y,z(Bohr): 21.149543 -8.793867 -3.157659
55(C) --> Charge: 6.000000 x,y,z(Bohr): 17.976836 -10.181159 -0.960449
56(C) --> Charge: 6.000000 x,y,z(Bohr): 15.844462 -9.383638   0.403230
57(H) --> Charge: 1.000000 x,y,z(Bohr): 14.604921 -10.761666   1.300678
58(C) --> Charge: 6.000000 x,y,z(Bohr): 15.240724 -6.824780   0.664135
59(H) --> Charge: 1.000000 x,y,z(Bohr): 13.565726 -6.286270   1.735240
60(C) --> Charge: 6.000000 x,y,z(Bohr): 18.695136 -12.977736 -1.295842
61(C) --> Charge: 6.000000 x,y,z(Bohr): 21.366722 -13.426276 -0.201700
62(H) --> Charge: 1.000000 x,y,z(Bohr): 22.799675 -12.258650 -1.156174
63(H) --> Charge: 1.000000 x,y,z(Bohr): 21.915772 -15.422457 -0.436777
64(H) --> Charge: 1.000000 x,y,z(Bohr): 21.419366 -12.974121   1.830690
65(C) --> Charge: 6.000000 x,y,z(Bohr): 18.690731 -13.641114 -4.141760
66(H) --> Charge: 1.000000 x,y,z(Bohr): 16.803817 -13.337649 -4.969475
67(H) --> Charge: 1.000000 x,y,z(Bohr): 19.205278 -15.641466 -4.416000
68(H) --> Charge: 1.000000 x,y,z(Bohr): 20.052864 -12.485138 -5.207617
69(C) --> Charge: 6.000000 x,y,z(Bohr): 16.843363 -14.764715   0.059414
70(H) --> Charge: 1.000000 x,y,z(Bohr): 16.804880 -14.425365   2.112687
71(H) --> Charge: 1.000000 x,y,z(Bohr): 17.430124 -16.738762 -0.241552
72(H) --> Charge: 1.000000 x,y,z(Bohr): 14.903872 -14.566444 -0.670131
73(C) --> Charge: 6.000000 x,y,z(Bohr): -1.123775 -1.854056 -0.296089
74(C) --> Charge: 6.000000 x,y,z(Bohr): -2.519884 -4.072817 -0.616503
75(H) --> Charge: 1.000000 x,y,z(Bohr): -1.560184 -5.871025 -0.819282
76(C) --> Charge: 6.000000 x,y,z(Bohr): -5.144482 -3.926707 -0.703782
77(H) --> Charge: 1.000000 x,y,z(Bohr): -6.234470 -5.656073 -0.973661
78(C) --> Charge: 6.000000 x,y,z(Bohr): -6.350930 -1.572651 -0.484450
79(C) --> Charge: 6.000000 x,y,z(Bohr): -4.805591   0.565769 -0.236440
80(H) --> Charge: 1.000000 x,y,z(Bohr): -5.623344   2.450109 -0.151685
81(C) --> Charge: 6.000000 x,y,z(Bohr): -9.086764 -1.199848 -0.550921
82(C) --> Charge: 6.000000 x,y,z(Bohr): -13.199729 -2.043652 -0.384558
83(C) --> Charge: 6.000000 x,y,z(Bohr): -12.748734   0.574011 -0.765441
84(C) --> Charge: 6.000000 x,y,z(Bohr): -14.780830   2.313374 -0.888490
85(C) --> Charge: 6.000000 x,y,z(Bohr): -17.195162   1.282377 -0.547633
86(H) --> Charge: 1.000000 x,y,z(Bohr): -18.834845   2.529635 -0.656881

87(C) --> Charge: 6.000000 x,y,z(Bohr): -17.603725 -1.317073 -0.147986
88(H) --> Charge: 1.000000 x,y,z(Bohr): -19.543019 -1.993045   0.047942
89(C) --> Charge: 6.000000 x,y,z(Bohr): -15.631042 -3.085311 -0.074386
90(C) --> Charge: 6.000000 x,y,z(Bohr): -16.065319 -5.819588   0.344518
91(C) --> Charge: 6.000000 x,y,z(Bohr): -14.758336 -7.666774 -1.046498
92(H) --> Charge: 1.000000 x,y,z(Bohr): -13.431703 -7.098027 -2.522610
93(C) --> Charge: 6.000000 x,y,z(Bohr): -15.181828 -10.244331 -0.641386
94(H) --> Charge: 1.000000 x,y,z(Bohr): -14.121115 -11.587176 -1.785935
95(C) --> Charge: 6.000000 x,y,z(Bohr): -16.929695 -11.097519   1.161751
96(C) --> Charge: 6.000000 x,y,z(Bohr): -18.247079 -9.235214   2.535409
97(H) --> Charge: 1.000000 x,y,z(Bohr): -19.624433 -9.796416   3.965258
98(C) --> Charge: 6.000000 x,y,z(Bohr): -17.833178 -6.665141   2.144302
99(H) --> Charge: 1.000000 x,y,z(Bohr): -18.866578 -5.286891   3.281079
100(C) --> Charge: 6.000000 x,y,z(Bohr): -17.439171 -13.913163   1.672869
101(C) --> Charge: 6.000000 x,y,z(Bohr): -20.255224 -14.493476   1.154309
102(H) --> Charge: 1.000000 x,y,z(Bohr): -20.653694 -16.505824   1.518228
103(H) --> Charge: 1.000000 x,y,z(Bohr): -20.742500 -14.083813 -0.828651
104(H) --> Charge: 1.000000 x,y,z(Bohr): -21.514338 -13.369368   2.370350
105(C) --> Charge: 6.000000 x,y,z(Bohr): -16.823964 -14.510507   4.465951
106(H) --> Charge: 1.000000 x,y,z(Bohr): -17.986746 -13.384779   5.773061
107(H) --> Charge: 1.000000 x,y,z(Bohr): -14.822106 -14.115820   4.883764
108(H) --> Charge: 1.000000 x,y,z(Bohr): -17.186418 -16.522436   4.867787
109(C) --> Charge: 6.000000 x,y,z(Bohr): -15.823816 -15.651042 -0.008544
110(H) --> Charge: 1.000000 x,y,z(Bohr): -13.787480 -15.364476   0.314185
111(H) --> Charge: 1.000000 x,y,z(Bohr): -16.212657 -15.350067 -2.031136
112(H) --> Charge: 1.000000 x,y,z(Bohr): -16.255686 -17.640006   0.425695
113(C) --> Charge: 6.000000 x,y,z(Bohr): -14.372580   5.046500 -1.339097
114(C) --> Charge: 6.000000 x,y,z(Bohr): -15.768660   6.878100 -0.022118
115(H) --> Charge: 1.000000 x,y,z(Bohr): -17.150863   6.282483   1.390773
116(C) --> Charge: 6.000000 x,y,z(Bohr): -15.383801   9.460418 -0.439966
117(H) --> Charge: 1.000000 x,y,z(Bohr): -16.503790 10.794667   0.657909
118(C) --> Charge: 6.000000 x,y,z(Bohr): -13.595503 10.330267 -2.194838
119(C) --> Charge: 6.000000 x,y,z(Bohr): -12.210703   8.480488 -3.519480
120(H) --> Charge: 1.000000 x,y,z(Bohr): -10.792817   9.055314 -4.903894
121(C) --> Charge: 6.000000 x,y,z(Bohr): -12.575619   5.907427 -3.105115
122(H) --> Charge: 1.000000 x,y,z(Bohr): -11.446480   4.537461 -4.150830
123(C) --> Charge: 6.000000 x,y,z(Bohr): -13.092460 13.151599 -2.686228
124(C) --> Charge: 6.000000 x,y,z(Bohr): -14.845382 14.873412 -1.131599
125(C) --> Charge: 6.000000 x,y,z(Bohr): -13.520676 13.726304 -5.519673
126(H) --> Charge: 1.000000 x,y,z(Bohr): -12.239193 12.627318 -6.734952
127(H) --> Charge: 1.000000 x,y,z(Bohr): -13.183149 15.745892 -5.904599
128(C) --> Charge: 6.000000 x,y,z(Bohr): -10.330977 13.784051 -1.974547
129(H) --> Charge: 1.000000 x,y,z(Bohr): -9.921862 15.785713 -2.383238
130(H) --> Charge: 1.000000 x,y,z(Bohr): -8.967247 12.627242 -3.036944
131(H) --> Charge: 1.000000 x,y,z(Bohr): -9.999794 13.466347   0.057639
132(H) --> Charge: 1.000000 x,y,z(Bohr): -15.476651 13.277890 -6.076754
133(H) --> Charge: 1.000000 x,y,z(Bohr): -14.418982 16.866693 -1.552145
134(H) --> Charge: 1.000000 x,y,z(Bohr): -16.850368 14.548956 -1.586927
135(H) --> Charge: 1.000000 x,y,z(Bohr): -14.587476 14.592338   0.914586
136(C) --> Charge: 6.000000 x,y,z(Bohr): -0.327263 -10.828286   1.387407
137(C) --> Charge: 6.000000 x,y,z(Bohr): -0.145044 -9.643471   3.981968
138(O) --> Charge: 8.000000 x,y,z(Bohr): -1.148943 -7.159429   3.865303
139(O) --> Charge: 8.000000 x,y,z(Bohr):   1.198965 -9.416757 -0.312826
140(C) --> Charge: 6.000000 x,y,z(Bohr): -1.504743 -6.059388   6.282776
141(C) --> Charge: 6.000000 x,y,z(Bohr): -2.764196 -3.497561   6.036580
142(O) --> Charge: 8.000000 x,y,z(Bohr): -0.960291 -1.607919   5.363828
143(C) --> Charge: 6.000000 x,y,z(Bohr): -1.642880   0.842643   6.207744
144(C) --> Charge: 6.000000 x,y,z(Bohr):   0.680464   2.484160   6.535514
145(O) --> Charge: 8.000000 x,y,z(Bohr):   1.317228   3.867165   4.277400
146(C) --> Charge: 6.000000 x,y,z(Bohr):   3.667658   5.193817   4.539194
147(C) --> Charge: 6.000000 x,y,z(Bohr):   3.668246   7.359322   2.682096
148(O) --> Charge: 8.000000 x,y,z(Bohr):   3.249746   6.317780   0.205352
149(C) --> Charge: 6.000000 x,y,z(Bohr):   2.893184   8.200763 -1.727793
150(C) --> Charge: 6.000000 x,y,z(Bohr):   1.216031 -10.358930 -2.830622
151(C) --> Charge: 6.000000 x,y,z(Bohr):   2.586396 -8.488094 -4.500198
152(O) --> Charge: 8.000000 x,y,z(Bohr):   1.053856 -6.302070 -4.786661
153(C) --> Charge: 6.000000 x,y,z(Bohr):   2.082880 -4.511322 -6.507618
154(C) --> Charge: 6.000000 x,y,z(Bohr):   0.091566 -2.567293 -7.218889
155(O) --> Charge: 8.000000 x,y,z(Bohr):   0.184975 -0.457400 -5.534758
156(C) --> Charge: 6.000000 x,y,z(Bohr): -1.461410   1.558046 -6.234487
157(C) --> Charge: 6.000000 x,y,z(Bohr):   0.011826   3.988214 -6.481857
158(O) --> Charge: 8.000000 x,y,z(Bohr):   0.757586   4.938314 -4.028631
159(C) --> Charge: 6.000000 x,y,z(Bohr):   2.710201   6.827285 -4.218730

160(H) --> Charge: 1.000000 x,y,z(Bohr): -2.320020 -10.819470   0.742732
161(H) --> Charge: 1.000000 x,y,z(Bohr):   0.314920 -12.816614   1.492863
162(H) --> Charge: 1.000000 x,y,z(Bohr): -1.231338 -10.819611   5.331523
163(H) --> Charge: 1.000000 x,y,z(Bohr):   1.850784 -9.612747   4.622161
164(H) --> Charge: 1.000000 x,y,z(Bohr): -2.722997 -7.295707   7.456055
165(H) --> Charge: 1.000000 x,y,z(Bohr):   0.322366 -5.828934   7.281187
166(H) --> Charge: 1.000000 x,y,z(Bohr): -4.302240 -3.593805   4.623422
167(H) --> Charge: 1.000000 x,y,z(Bohr): -3.620241 -3.020580   7.882696
168(H) --> Charge: 1.000000 x,y,z(Bohr): -3.002808   1.754750   4.904172
169(H) --> Charge: 1.000000 x,y,z(Bohr): -2.583803   0.701582   8.068977
170(H) --> Charge: 1.000000 x,y,z(Bohr):   0.334539   3.878232   8.048884
171(H) --> Charge: 1.000000 x,y,z(Bohr):   2.281180   1.271547   7.100314
172(H) --> Charge: 1.000000 x,y,z(Bohr):   3.861651   5.970329   6.463358
173(H) --> Charge: 1.000000 x,y,z(Bohr):   5.256240   3.884118   4.201190
174(H) --> Charge: 1.000000 x,y,z(Bohr):   2.145411   8.711894   3.131195
175(H) --> Charge: 1.000000 x,y,z(Bohr):   5.496713   8.356988   2.716655
176(H) --> Charge: 1.000000 x,y,z(Bohr):   1.145634   9.262434 -1.324565
177(H) --> Charge: 1.000000 x,y,z(Bohr):   4.520046   9.501052 -1.758040
178(H) --> Charge: 1.000000 x,y,z(Bohr): -0.738868 -10.617578 -3.533669
179(H) --> Charge: 1.000000 x,y,z(Bohr):   2.190890 -12.208120 -2.908396
180(H) --> Charge: 1.000000 x,y,z(Bohr):   2.940219 -9.379227 -6.360860
181(H) --> Charge: 1.000000 x,y,z(Bohr):   4.444655 -8.001519 -3.665561
182(H) --> Charge: 1.000000 x,y,z(Bohr):   2.722671 -5.488104 -8.243013
183(H) --> Charge: 1.000000 x,y,z(Bohr):   3.739166 -3.541368 -5.670322
184(H) --> Charge: 1.000000 x,y,z(Bohr): -1.786558 -3.485561 -7.170136
185(H) --> Charge: 1.000000 x,y,z(Bohr):   0.446654 -1.911174 -9.170552
186(H) --> Charge: 1.000000 x,y,z(Bohr): -2.988402   1.756118 -4.826756
187(H) --> Charge: 1.000000 x,y,z(Bohr): -2.360216   1.196020 -8.079563
188(H) --> Charge: 1.000000 x,y,z(Bohr): -1.153976   5.447415 -7.411091
189(H) --> Charge: 1.000000 x,y,z(Bohr):   1.719513   3.642410 -7.625996
190(H) --> Charge: 1.000000 x,y,z(Bohr):   2.227534   8.191108 -5.717647
191(H) --> Charge: 1.000000 x,y,z(Bohr):   4.512155   5.892452 -4.699411
192(O) --> Charge: 8.000000 x,y,z(Bohr): -2.570756   6.094481   0.506027
193(C) --> Charge: 6.000000 x,y,z(Bohr): -3.416658   6.797789   2.627855
194(H) --> Charge: 1.000000 x,y,z(Bohr): -2.183414   6.836805   4.298833
195(N) --> Charge: 7.000000 x,y,z(Bohr): -5.785818   7.485483   3.017013
196(C) --> Charge: 6.000000 x,y,z(Bohr): -6.675437   8.179979   5.536039
197(C) --> Charge: 6.000000 x,y,z(Bohr): -7.675773   7.408332   0.999770
198(H) --> Charge: 1.000000 x,y,z(Bohr): -8.209971   6.897014   6.108143
199(H) --> Charge: 1.000000 x,y,z(Bohr): -5.121762   8.037495   6.902867
200(H) --> Charge: 1.000000 x,y,z(Bohr): -7.402954 10.127778   5.513742
201(H) --> Charge: 1.000000 x,y,z(Bohr): -8.832363   5.686068   1.163713
202(H) --> Charge: 1.000000 x,y,z(Bohr): -8.906392   9.069406   1.157507
203(H) --> Charge: 1.000000 x,y,z(Bohr): -6.727564   7.415322 -0.839412

Note:

Orbital   370 is HOMO, energy: -0.215634 a.u.   -5.867692 eV

Orbital   371 is LUMO, energy: -0.105282 a.u.   -2.864867 eV

HOMO-LUMO gap: 0.110352 a.u.   3.002825 eV   289.728619 kJ/mol

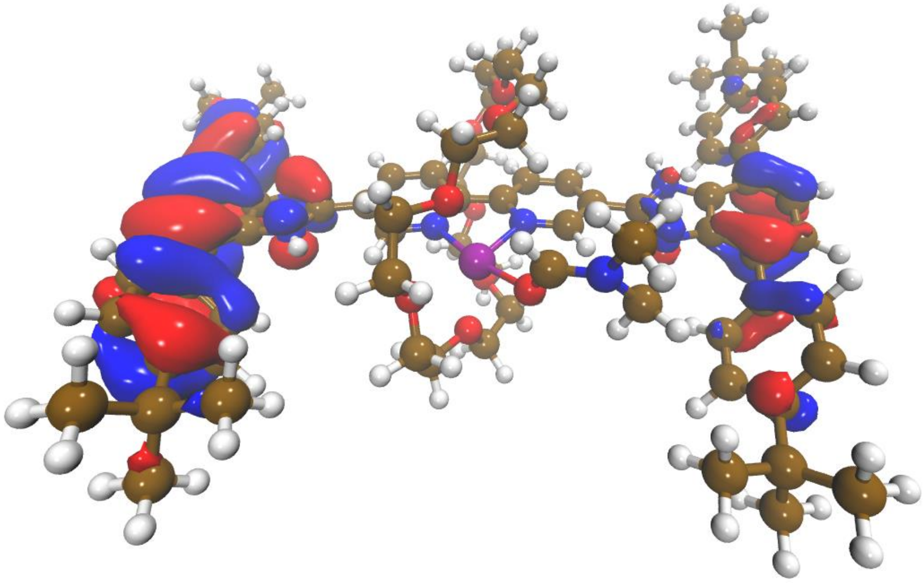

Figure S3. Highest occupied molecular orbital (HOMO) of the optimized [2]molecular shuttle with Zn(II) calculated at C-PCM(DMF)/B3LYP(D3)/cc-pVTZ level of theory. The blue colour reflects the positive (+) part of the corresponding eigenvector and an isodensity value of 0.015 $e{\cdot}a_0^3$ is used.

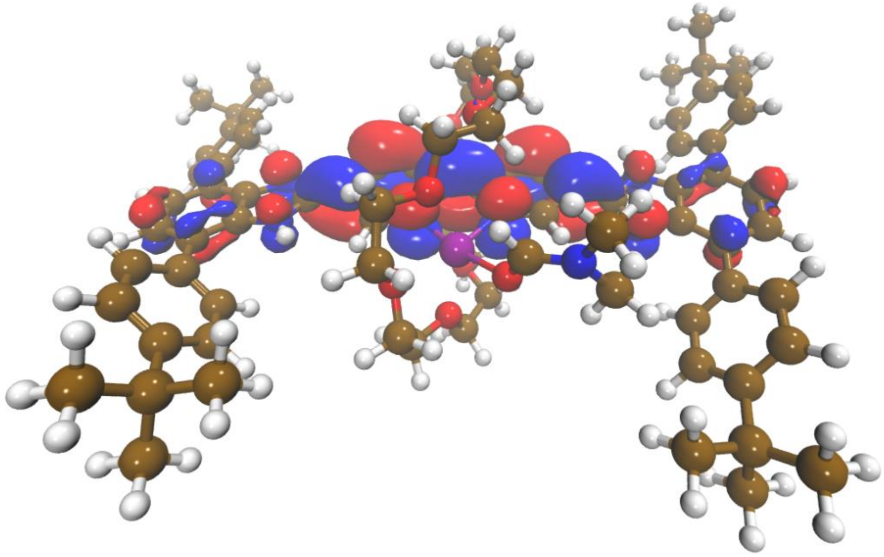

Figure S4. Lowest unoccupied molecular orbital (LUMO) of the optimized [2]molecular shuttle with Zn(II) calculated at C-PCM(DMF)/B3LYP(D3)/cc-pVTZ level of theory. The blue colour reflects the positive (+) part of the corresponding eigenvector and an isodensity value of 0.015 $e{\cdot}a_0^3$ is used.

Fuzzy Bond Order analysis (FBO) - as extracted considering the optimized [2]molecular shuttle with Zn(II) at C-PCM(DMF)/B3LYP(D3)/cc-pVTZ level of theory - showing the interatomic couples featuring a total bond order >= 0.050000.

1: 1(Zn) 2(N) 0,71823313 (equatorial position)
2: 1(Zn) 3(N) 0,70744865 (equatorial position)
3: 1(Zn) 10(C) 0,05706881
4: 1(Zn) 16(C) 0,05788693
5: 1(Zn) 73(C) 0,05541509
6: 1(Zn) 79(C) 0,05635572
7: 1(Zn) 145(O) 0,38618599 (axial MAC)

#	8:	1(Zn)	148(O)	0,56106884	(equatorial MAC)	
#	9:	1(Zn)	158(O)	0,59131651	(axial MAC)	
#	10:	1(Zn)	192(O)	0,80277376	(equatorial DMF)	
#	11:	1(Zn)	193(C)	0,07702422		
#	12:	2(N)	10(C)	1,34814777		
#	13:	2(N)	11(C)	0,08682786		
#	14:	2(N)	13(C)	0,08271331		
#	15:	2(N)	15(C)	0,09236942		
#	16:	2(N)	16(C)	1,45891186		
#	17:	2(N)	73(C)	0,06430486		
#	18:	3(N)	10(C)	0,06406243		
#	19:	3(N)	73(C)	1,33644723		
#	20:	3(N)	74(C)	0,08473812		
#	21:	3(N)	76(C)	0,08060954		
#	22:	3(N)	78(C)	0,09191032		
#	23:	3(N)	79(C)	1,46675339		
#	24:	4(N)	5(N)	0,16531982		
#	25:	4(N)	78(C)	0,07605247		
#	26:	4(N)	79(C)	0,05635142		
#	27:	4(N)	81(C)	1,55833306		
#	28:	4(N)	82(C)	0,09803799		
#	29:	4(N)	83(C)	1,32561701		
#	30:	4(N)	84(C)	0,06860078		
#	31:	4(N)	121(C)	0,05154512		
#	32:	5(N)	6(H)	0,80065711		
#	33:	5(N)	78(C)	0,06464442		
#	34:	5(N)	81(C)	1,30264794		
#	35:	5(N)	82(C)	1,26604033		
#	36:	5(N)	83(C)	0,10926956		
#	37:	5(N)	89(C)	0,07203777		
#	38:	7(N)	8(H)	0,79109229		
#	39:	7(N)	9(N)	0,16572593		
#	40:	7(N)	15(C)	0,06652965		
#	41:	7(N)	18(C)	1,29201429		
#	42:	7(N)	19(C)	1,26814221		
#	43:	7(N)	20(C)	0,10986769		
#	44:	7(N)	26(C)	0,07286458		
#	45:	9(N)	15(C)	0,07520178		
#	46:	9(N)	18(C)	1,57108850		
#	47:	9(N)	19(C)	0,09765923		
#	48:	9(N)	20(C)	1,32512587		
#	49:	9(N)	21(C)	0,06725404		
#	50:	9(N)	58(C)	0,05822383		
#	51:	10(C)	11(C)	1,33745753		
#	52:	10(C)	13(C)	0,10339320		
#	53:	10(C)	15(C)	0,07696386		
#	54:	10(C)	16(C)	0,10335919		
#	55:	10(C)	73(C)	1,03737238		
#	56:	10(C)	74(C)	0,05995059		
#	57:	11(C)	12(H)	0,74946518		
#	58:	11(C)	13(C)	1,45373344		
#	59:	11(C)	15(C)	0,09654241		
#	60:	11(C)	16(C)	0,08603759		
#	61:	11(C)	73(C)	0,06073342		
#	62:	11(C)	139(O)	0,06928720		
#	63:	12(H)	139(O)	0,05862489		
#	64:	13(C)	14(H)	0,83928292		
#	65:	13(C)	15(C)	1,33934431		
#	66:	13(C)	16(C)	0,09535377		
#	67:	13(C)	18(C)	0,06786040		
#	68:	15(C)	16(C)	1,34292752		
#	69:	15(C)	18(C)	1,09430996		
#	70:	16(C)	17(H)	0,78725483		
#	71:	16(C)	18(C)	0,07223537		
#	72:	16(C)	148(O)	0,05086008		
#	73:	18(C)	19(C)	0,11413720		
#	74:	18(C)	20(C)	0,13090762		
#	75:	19(C)	20(C)	1,22478430		
#	76:	19(C)	21(C)	0,07624454		
#	77:	19(C)	22(C)	0,06851279		
#	78:	19(C)	24(C)	0,09885985		
#	79:	19(C)	26(C)	1,28337777		
#	80:	19(C)	27(C)	0,06761538		

#	81:	20(C)	21(C)	1,25881438
#	82:	20(C)	22(C)	0,09330100
#	83:	20(C)	24(C)	0,06641433
#	84:	20(C)	26(C)	0,06823642
#	85:	20(C)	50(C)	0,06304378
#	86:	21(C)	22(C)	1,41092625
#	87:	21(C)	24(C)	0,09294595
#	88:	21(C)	26(C)	0,07839164
#	89:	21(C)	50(C)	1,08895626
#	90:	21(C)	51(C)	0,07702576
#	91:	21(C)	58(C)	0,07393111
#	92:	22(C)	23(H)	0,85188974
#	93:	22(C)	24(C)	1,42400035
#	94:	22(C)	26(C)	0,09412908
#	95:	22(C)	50(C)	0,07681019
#	96:	24(C)	25(H)	0,85942153
#	97:	24(C)	26(C)	1,40961131
#	98:	24(C)	27(C)	0,07305976
#	99:	26(C)	27(C)	1,07916995
#	100:	26(C)	28(C)	0,07868917
#	101:	26(C)	35(C)	0,07575342
#	102:	27(C)	28(C)	1,38515298
#	103:	27(C)	30(C)	0,09787156
#	104:	27(C)	32(C)	0,08596715
#	105:	27(C)	33(C)	0,09657349
#	106:	27(C)	35(C)	1,35790036
#	107:	28(C)	29(H)	0,85822427
#	108:	28(C)	30(C)	1,45596731
#	109:	28(C)	32(C)	0,09763584
#	110:	28(C)	33(C)	0,09986509
#	111:	28(C)	35(C)	0,09639775
#	112:	30(C)	31(H)	0,84441032
#	113:	30(C)	32(C)	1,39746589
#	114:	30(C)	33(C)	0,10088575
#	115:	30(C)	35(C)	0,09668486
#	116:	30(C)	37(C)	0,06158468
#	117:	30(C)	42(C)	0,05486693
#	118:	32(C)	33(C)	1,37123710
#	119:	32(C)	35(C)	0,09592515
#	120:	32(C)	37(C)	0,99242607
#	121:	32(C)	38(C)	0,08087816
#	122:	32(C)	42(C)	0,06502535
#	123:	32(C)	46(C)	0,08337565
#	124:	33(C)	34(H)	0,85521027
#	125:	33(C)	35(C)	1,47726276
#	126:	33(C)	37(C)	0,06532323
#	127:	35(C)	36(H)	0,84623158
#	128:	37(C)	38(C)	1,04407531
#	129:	37(C)	42(C)	1,06087545
#	130:	37(C)	46(C)	1,04797517
#	131:	38(C)	39(H)	0,88890663
#	132:	38(C)	40(H)	0,87960735
#	133:	38(C)	41(H)	0,89249863
#	134:	38(C)	42(C)	0,09047030
#	135:	38(C)	46(C)	0,08572812
#	136:	42(C)	43(H)	0,89045030
#	137:	42(C)	44(H)	0,88485291
#	138:	42(C)	45(H)	0,88451227
#	139:	42(C)	46(C)	0,09155626
#	140:	46(C)	47(H)	0,88467710
#	141:	46(C)	48(H)	0,88933897
#	142:	46(C)	49(H)	0,89181655
#	143:	50(C)	51(C)	1,36035972
#	144:	50(C)	53(C)	0,09817314
#	145:	50(C)	55(C)	0,08526733
#	146:	50(C)	56(C)	0,09710575
#	147:	50(C)	58(C)	1,37176271
#	148:	51(C)	52(H)	0,85555514
#	149:	51(C)	53(C)	1,48396999
#	150:	51(C)	55(C)	0,09703087
#	151:	51(C)	56(C)	0,09640082
#	152:	51(C)	58(C)	0,09602037
#	153:	53(C)	54(H)	0,85620342

# 154:	53(C)	55(C)	1,37229898
# 155:	53(C)	56(C)	0,10060546
# 156:	53(C)	58(C)	0,09907667
# 157:	53(C)	60(C)	0,06618423
# 158:	55(C)	56(C)	1,39746672
# 159:	55(C)	58(C)	0,09771654
# 160:	55(C)	60(C)	0,99357823
# 161:	55(C)	61(C)	0,08271333
# 162:	55(C)	65(C)	0,08306970
# 163:	55(C)	69(C)	0,06484781
# 164:	56(C)	57(H)	0,84602988
# 165:	56(C)	58(C)	1,45937722
# 166:	56(C)	60(C)	0,06209950
# 167:	56(C)	69(C)	0,05653134
# 168:	58(C)	59(H)	0,83084512
# 169:	60(C)	61(C)	1,04890029
# 170:	60(C)	65(C)	1,04837301
# 171:	60(C)	69(C)	1,06105099
# 172:	61(C)	62(H)	0,88328472
# 173:	61(C)	63(H)	0,89086088
# 174:	61(C)	64(H)	0,88918909
# 175:	61(C)	65(C)	0,08732420
# 176:	61(C)	69(C)	0,09204680
# 177:	65(C)	66(H)	0,89089316
# 178:	65(C)	67(H)	0,89239641
# 179:	65(C)	68(H)	0,88274217
# 180:	65(C)	69(C)	0,09195162
# 181:	69(C)	70(H)	0,88561236
# 182:	69(C)	71(H)	0,89055248
# 183:	69(C)	72(H)	0,88642813
# 184:	73(C)	74(C)	1,33338414
# 185:	73(C)	76(C)	0,10227689
# 186:	73(C)	78(C)	0,07597451
# 187:	73(C)	79(C)	0,10271305
# 188:	74(C)	75(H)	0,76834135
# 189:	74(C)	76(C)	1,44045814
# 190:	74(C)	78(C)	0,09459412
# 191:	74(C)	79(C)	0,08708281
# 192:	74(C)	138(O)	0,05662338
# 193:	76(C)	77(H)	0,84932472
# 194:	76(C)	78(C)	1,35241599
# 195:	76(C)	79(C)	0,09443868
# 196:	76(C)	81(C)	0,06686041
# 197:	78(C)	79(C)	1,33007716
# 198:	78(C)	81(C)	1,09266762
# 199:	79(C)	80(H)	0,79220840
# 200:	79(C)	81(C)	0,07333314
# 201:	81(C)	82(C)	0,11490435
# 202:	81(C)	83(C)	0,12927001
# 203:	82(C)	83(C)	1,22419215
# 204:	82(C)	84(C)	0,07448388
# 205:	82(C)	85(C)	0,06791787
# 206:	82(C)	87(C)	0,09817251
# 207:	82(C)	89(C)	1,28570185
# 208:	82(C)	90(C)	0,06595788
# 209:	83(C)	84(C)	1,25827487
# 210:	83(C)	85(C)	0,09385941
# 211:	83(C)	87(C)	0,06714092
# 212:	83(C)	89(C)	0,06976772
# 213:	83(C)	113(C)	0,06310554
# 214:	84(C)	85(C)	1,41525006
# 215:	84(C)	87(C)	0,09378383
# 216:	84(C)	89(C)	0,07851149
# 217:	84(C)	113(C)	1,08196127
# 218:	84(C)	114(C)	0,07832837
# 219:	84(C)	121(C)	0,07268587
# 220:	85(C)	86(H)	0,85504891
# 221:	85(C)	87(C)	1,42363027
# 222:	85(C)	89(C)	0,09314461
# 223:	85(C)	113(C)	0,07694357
# 224:	87(C)	88(H)	0,85385052
# 225:	87(C)	89(C)	1,40463886
# 226:	87(C)	90(C)	0,07532587

#	227:	89(C)	90(C)	1,08287934
#	228:	89(C)	91(C)	0,07696823
#	229:	89(C)	98(C)	0,07780776
#	230:	90(C)	91(C)	1,38009773
#	231:	90(C)	93(C)	0,09713813
#	232:	90(C)	95(C)	0,08573320
#	233:	90(C)	96(C)	0,09856192
#	234:	90(C)	98(C)	1,36220307
#	235:	91(C)	92(H)	0,84690912
#	236:	91(C)	93(C)	1,45649897
#	237:	91(C)	95(C)	0,09765468
#	238:	91(C)	96(C)	0,09979883
#	239:	91(C)	98(C)	0,09627277
#	240:	93(C)	94(H)	0,84500090
#	241:	93(C)	95(C)	1,39962093
#	242:	93(C)	96(C)	0,10114808
#	243:	93(C)	98(C)	0,09696743
#	244:	93(C)	100(C)	0,06215855
#	245:	93(C)	109(C)	0,05696867
#	246:	95(C)	96(C)	1,37007170
#	247:	95(C)	98(C)	0,09685236
#	248:	95(C)	100(C)	0,99338684
#	249:	95(C)	101(C)	0,08300984
#	250:	95(C)	105(C)	0,08309631
#	251:	95(C)	109(C)	0,06503074
#	252:	96(C)	97(H)	0,85406049
#	253:	96(C)	98(C)	1,48271603
#	254:	96(C)	100(C)	0,06586181
#	255:	98(C)	99(H)	0,85631921
#	256:	100(C)	101(C)	1,04863877
#	257:	100(C)	105(C)	1,04827549
#	258:	100(C)	109(C)	1,06096447
#	259:	101(C)	102(H)	0,89092838
#	260:	101(C)	103(H)	0,88856527
#	261:	101(C)	104(H)	0,88289105
#	262:	101(C)	105(C)	0,08706439
#	263:	101(C)	109(C)	0,09180573
#	264:	105(C)	106(H)	0,88320936
#	265:	105(C)	107(H)	0,88965617
#	266:	105(C)	108(H)	0,89090605
#	267:	105(C)	109(C)	0,09179313
#	268:	109(C)	110(H)	0,88619063
#	269:	109(C)	111(H)	0,88554664
#	270:	109(C)	112(H)	0,88998809
#	271:	113(C)	114(C)	1,38209651
#	272:	113(C)	116(C)	0,09736368
#	273:	113(C)	118(C)	0,08560038
#	274:	113(C)	119(C)	0,09588779
#	275:	113(C)	121(C)	1,35092436
#	276:	114(C)	115(H)	0,85604709
#	277:	114(C)	116(C)	1,45641777
#	278:	114(C)	118(C)	0,09751980
#	279:	114(C)	119(C)	0,09887202
#	280:	114(C)	121(C)	0,09552110
#	281:	116(C)	117(H)	0,84521448
#	282:	116(C)	118(C)	1,40066933
#	283:	116(C)	119(C)	0,09976910
#	284:	116(C)	121(C)	0,09603106
#	285:	116(C)	123(C)	0,06196686
#	286:	116(C)	124(C)	0,05681907
#	287:	118(C)	119(C)	1,36711770
#	288:	118(C)	121(C)	0,09553843
#	289:	118(C)	123(C)	0,99271282
#	290:	118(C)	124(C)	0,06487475
#	291:	118(C)	125(C)	0,08235330
#	292:	118(C)	128(C)	0,08091989
#	293:	119(C)	120(H)	0,85356208
#	294:	119(C)	121(C)	1,47523073
#	295:	119(C)	123(C)	0,06496528
#	296:	121(C)	122(H)	0,83694663
#	297:	123(C)	124(C)	1,06036348
#	298:	123(C)	125(C)	1,04764215
#	299:	123(C)	128(C)	1,04684491

#	300:	124(C)	125(C)	0,09175075
#	301:	124(C)	128(C)	0,09112494
#	302:	124(C)	133(H)	0,88996927
#	303:	124(C)	134(H)	0,88528587
#	304:	124(C)	135(H)	0,88571535
#	305:	125(C)	126(H)	0,88264074
#	306:	125(C)	127(H)	0,89129325
#	307:	125(C)	128(C)	0,08633011
#	308:	125(C)	132(H)	0,88831574
#	309:	128(C)	129(H)	0,89126211
#	310:	128(C)	130(H)	0,88323609
#	311:	128(C)	131(H)	0,88585562
#	312:	136(C)	137(C)	1,08059030
#	313:	136(C)	138(O)	0,09384162
#	314:	136(C)	139(O)	1,19727192
#	315:	136(C)	150(C)	0,08994360
#	316:	136(C)	160(H)	0,83141892
#	317:	136(C)	161(H)	0,84189418
#	318:	137(C)	138(O)	1,20234321
#	319:	137(C)	139(O)	0,09263495
#	320:	137(C)	140(C)	0,09460679
#	321:	137(C)	162(H)	0,83780778
#	322:	137(C)	163(H)	0,83476654
#	323:	138(O)	140(C)	1,20596431
#	324:	138(O)	141(C)	0,09081642
#	325:	138(O)	162(H)	0,05877287
#	326:	138(O)	163(H)	0,06090416
#	327:	138(O)	164(H)	0,06223004
#	328:	138(O)	165(H)	0,05937161
#	329:	139(O)	150(C)	1,19536113
#	330:	139(O)	151(C)	0,09428019
#	331:	139(O)	160(H)	0,05984156
#	332:	139(O)	161(H)	0,05894159
#	333:	139(O)	178(H)	0,05808976
#	334:	139(O)	179(H)	0,06045231
#	335:	140(C)	141(C)	1,07371403
#	336:	140(C)	142(O)	0,09561719
#	337:	140(C)	164(H)	0,83834343
#	338:	140(C)	165(H)	0,82774864
#	339:	141(C)	142(O)	1,18468236
#	340:	141(C)	143(C)	0,09195820
#	341:	141(C)	166(H)	0,81238213
#	342:	141(C)	167(H)	0,83515279
#	343:	142(O)	143(C)	1,20172347
#	344:	142(O)	144(C)	0,09789591
#	345:	142(O)	166(H)	0,05309727
#	346:	142(O)	167(H)	0,05634083
#	347:	142(O)	168(H)	0,05139488
#	348:	142(O)	169(H)	0,05756740
#	349:	143(C)	144(C)	1,06987102
#	350:	143(C)	145(O)	0,07999043
#	351:	143(C)	168(H)	0,80578517
#	352:	143(C)	169(H)	0,83252365
#	353:	144(C)	145(O)	1,13252888
#	354:	144(C)	146(C)	0,09113774
#	355:	144(C)	170(H)	0,83971039
#	356:	144(C)	171(H)	0,81938527
#	357:	145(O)	146(C)	1,13649503
#	358:	145(O)	147(C)	0,08554871
#	359:	145(O)	170(H)	0,05865728
#	360:	145(O)	171(H)	0,05289602
#	361:	145(O)	173(H)	0,05497668
#	362:	146(C)	147(C)	1,07222197
#	363:	146(C)	148(O)	0,07977459
#	364:	146(C)	172(H)	0,83089297
#	365:	146(C)	173(H)	0,82325342
#	366:	147(C)	148(O)	1,10806820
#	367:	147(C)	149(C)	0,07871134
#	368:	147(C)	174(H)	0,82619305
#	369:	147(C)	175(H)	0,83893606
#	370:	148(O)	149(C)	1,10060661
#	371:	148(O)	159(C)	0,08250442
#	372:	148(O)	174(H)	0,05372213

# 373:	148(O)	176(H)	0,05316727
# 374:	149(C)	158(O)	0,07579925
# 375:	149(C)	159(C)	1,07294280
# 376:	149(C)	176(H)	0,81893665
# 377:	149(C)	177(H)	0,83915118
# 378:	150(C)	151(C)	1,07732326
# 379:	150(C)	152(O)	0,09656757
# 380:	150(C)	178(H)	0,82886856
# 381:	150(C)	179(H)	0,84375533
# 382:	151(C)	152(O)	1,20063664
# 383:	151(C)	153(C)	0,09268690
# 384:	151(C)	180(H)	0,83943820
# 385:	151(C)	181(H)	0,82374204
# 386:	152(O)	153(C)	1,19863547
# 387:	152(O)	154(C)	0,09892956
# 388:	152(O)	180(H)	0,05983151
# 389:	152(O)	181(H)	0,05707478
# 390:	152(O)	182(H)	0,05993301
# 391:	152(O)	183(H)	0,05522231
# 392:	153(C)	154(C)	1,06618490
# 393:	153(C)	155(O)	0,09516180
# 394:	153(C)	182(H)	0,83830542
# 395:	153(C)	183(H)	0,81497910
# 396:	154(C)	155(O)	1,18817922
# 397:	154(C)	156(C)	0,09846041
# 398:	154(C)	184(H)	0,82490758
# 399:	154(C)	185(H)	0,84110895
# 400:	155(O)	156(C)	1,17902555
# 401:	155(O)	157(C)	0,10153274
# 402:	155(O)	184(H)	0,05642306
# 403:	155(O)	185(H)	0,06066383
# 404:	156(C)	157(C)	1,07894711
# 405:	156(C)	158(O)	0,07744038
# 406:	156(C)	186(H)	0,80261453
# 407:	156(C)	187(H)	0,82888418
# 408:	157(C)	158(O)	1,10342670
# 409:	157(C)	159(C)	0,08824872
# 410:	157(C)	188(H)	0,84247337
# 411:	157(C)	189(H)	0,82183037
# 412:	158(O)	159(C)	1,11590019
# 413:	158(O)	188(H)	0,05689997
# 414:	158(O)	189(H)	0,05048675
# 415:	158(O)	191(H)	0,05747057
# 416:	159(C)	190(H)	0,83283852
# 417:	159(C)	191(H)	0,83384022
# 418:	192(O)	193(C)	1,62977888
# 419:	192(O)	194(H)	0,05513904
# 420:	192(O)	195(N)	0,19330251
# 421:	192(O)	197(C)	0,06624250
# 422:	193(C)	194(H)	0,78213271
# 423:	193(C)	195(N)	1,53337304
# 424:	193(C)	196(C)	0,09286676
# 425:	193(C)	197(C)	0,07887716
# 426:	195(N)	196(C)	1,14259591
# 427:	195(N)	197(C)	1,12656774
# 428:	195(N)	198(H)	0,05198983
# 429:	195(N)	200(H)	0,05244892
# 430:	196(C)	197(C)	0,07591605
# 431:	196(C)	198(H)	0,87590947
# 432:	196(C)	199(H)	0,87815835
# 433:	196(C)	200(H)	0,87967326
# 434:	197(C)	201(H)	0,82694112
# 435:	197(C)	202(H)	0,84552674
# 436:	197(C)	203(H)	0,84140013

Optimized structure - at C-PCM(DMF)/B3LYP(D3)/cc-pVTZ level of theory – of the Stop-[Bzi-Bipy-Bzi]-Stop molecular thread in the *trans* conformation (see Figure 3, left panel in the manuscript):

1(N) --> Charge: 7.000000 x,y,z(Bohr): -10.829861 2.101713 0.000102
2(N) --> Charge: 7.000000 x,y,z(Bohr): -10.826681 -2.133769 0.008447

3(H) --> Charge: 1.000000 x,y,z(Bohr): -10.178474 -3.929638 -0.106838
4(N) --> Charge: 7.000000 x,y,z(Bohr): -2.654081 -2.170295 0.112344
5(N) --> Charge: 7.000000 x,y,z(Bohr): 2.628241 2.172881 -0.128650
6(N) --> Charge: 7.000000 x,y,z(Bohr): 10.800949 2.134454 -0.019944
7(H) --> Charge: 1.000000 x,y,z(Bohr): 10.154153 3.929525 0.113762
8(N) --> Charge: 7.000000 x,y,z(Bohr): 10.803087 -2.100939 -0.038919
9(C) --> Charge: 6.000000 x,y,z(Bohr): -1.416440 0.053716 -0.005792
10(C) --> Charge: 6.000000 x,y,z(Bohr): 15.913381 -14.688026 -2.849173
11(H) --> Charge: 1.000000 x,y,z(Bohr): 14.099331 -14.133093 -3.708876
12(H) --> Charge: 1.000000 x,y,z(Bohr): 16.056579 -16.765607 -2.920099
13(H) --> Charge: 1.000000 x,y,z(Bohr): 17.444580 -13.907469 -4.021393
14(C) --> Charge: 6.000000 x,y,z(Bohr): -2.706221 2.377359 -0.115328
15(H) --> Charge: 1.000000 x,y,z(Bohr): -1.627251 4.126655 -0.202738
16(C) --> Charge: 6.000000 x,y,z(Bohr): -5.328246 2.384388 -0.113085
17(H) --> Charge: 1.000000 x,y,z(Bohr): -6.392553 4.146629 -0.195373
18(C) --> Charge: 6.000000 x,y,z(Bohr): -6.638534 0.074005 -0.000807
19(C) --> Charge: 6.000000 x,y,z(Bohr): -5.172756 -2.142430 0.117011
20(H) --> Charge: 1.000000 x,y,z(Bohr): -6.072663 -4.000901 0.229796
21(C) --> Charge: 6.000000 x,y,z(Bohr): -9.402999 0.044786 -0.000837
22(C) --> Charge: 6.000000 x,y,z(Bohr): -13.303078 1.251073 -0.015056
23(C) --> Charge: 6.000000 x,y,z(Bohr): -13.340623 -1.433460 0.004431
24(C) --> Charge: 6.000000 x,y,z(Bohr): -15.571021 -2.883692 -0.017703
25(C) --> Charge: 6.000000 x,y,z(Bohr): -17.806746 -1.462973 -0.077555
26(H) --> Charge: 1.000000 x,y,z(Bohr): -19.613257 -2.459556 -0.088076
27(C) --> Charge: 6.000000 x,y,z(Bohr): -17.814293 1.195371 -0.085849
28(H) --> Charge: 1.000000 x,y,z(Bohr): -19.635127 2.161544 -0.156306
29(C) --> Charge: 6.000000 x,y,z(Bohr): -15.593660 2.644798 -0.048074
30(C) --> Charge: 6.000000 x,y,z(Bohr): -13.865627 6.903972 -1.332941
31(H) --> Charge: 1.000000 x,y,z(Bohr): -12.341906 5.960695 -2.346221
32(C) --> Charge: 6.000000 x,y,z(Bohr): -13.993426 9.543204 -1.349326
33(H) --> Charge: 1.000000 x,y,z(Bohr): -12.547419 10.569762 -2.396465
34(C) --> Charge: 6.000000 x,y,z(Bohr): -15.913979 10.856690 -0.074382
35(C) --> Charge: 6.000000 x,y,z(Bohr): -17.710887 9.385951 1.227044
36(H) --> Charge: 1.000000 x,y,z(Bohr): -19.234756 10.308012 2.268861
37(C) --> Charge: 6.000000 x,y,z(Bohr): -17.598726 6.755541 1.247858
38(H) --> Charge: 1.000000 x,y,z(Bohr): -19.011418 5.700951 2.320918
39(C) --> Charge: 6.000000 x,y,z(Bohr): -15.670733 5.444691 -0.040650
40(C) --> Charge: 6.000000 x,y,z(Bohr): -16.109720 13.756562 -0.044690
41(C) --> Charge: 6.000000 x,y,z(Bohr): -15.946217 14.707319 2.713048
42(H) --> Charge: 1.000000 x,y,z(Bohr): -17.478213 13.934724 3.889491
43(H) --> Charge: 1.000000 x,y,z(Bohr): -16.090206 16.785303 2.768845
44(H) --> Charge: 1.000000 x,y,z(Bohr): -14.132749 14.159253 3.578368
45(C) --> Charge: 6.000000 x,y,z(Bohr): -18.670696 14.562909 -1.196140
46(H) --> Charge: 1.000000 x,y,z(Bohr): -18.833548 13.910978 -3.167522
47(H) --> Charge: 1.000000 x,y,z(Bohr): -18.845577 16.639228 -1.182743
48(H) --> Charge: 1.000000 x,y,z(Bohr): -20.277825 13.785307 -0.128240
49(C) --> Charge: 6.000000 x,y,z(Bohr): -13.983967 15.022610 -1.572569
50(H) --> Charge: 1.000000 x,y,z(Bohr): -12.103446 14.561698 -0.808506
51(H) --> Charge: 1.000000 x,y,z(Bohr): -14.204944 17.090787 -1.496791
52(H) --> Charge: 1.000000 x,y,z(Bohr): -14.029618 14.457144 -3.575302
53(C) --> Charge: 6.000000 x,y,z(Bohr): -15.559275 -5.683492 -0.001668
54(C) --> Charge: 6.000000 x,y,z(Bohr): -17.210742 -7.071044 -1.547212
55(H) --> Charge: 1.000000 x,y,z(Bohr): -18.493419 -6.071151 -2.818151
56(C) --> Charge: 6.000000 x,y,z(Bohr): -17.212861 -9.713003 -1.525583

57(H) --> Charge: 1.000000 x,y,z(Bohr): -18.527085 -10.686978 -2.776038
58(C) --> Charge: 6.000000 x,y,z(Bohr): -15.568293 -11.093633   0.033951
59(C) --> Charge: 6.000000 x,y,z(Bohr): -13.923206 -9.691504   1.586231
60(H) --> Charge: 1.000000 x,y,z(Bohr): -12.620892 -10.668146   2.853502
61(C) --> Charge: 6.000000 x,y,z(Bohr): -13.913806 -7.057314   1.577588
62(H) --> Charge: 1.000000 x,y,z(Bohr): -12.647906 -6.055606   2.864765
63(C) --> Charge: 6.000000 x,y,z(Bohr): -15.507548 -13.998940   0.101743
64(C) --> Charge: 6.000000 x,y,z(Bohr):   1.390460 -0.051209 -0.013182
65(C) --> Charge: 6.000000 x,y,z(Bohr):   2.679871 -2.375131   0.092506
66(H) --> Charge: 1.000000 x,y,z(Bohr):   1.600520 -4.124271   0.177327
67(C) --> Charge: 6.000000 x,y,z(Bohr):   5.301793 -2.382648   0.088909
68(H) --> Charge: 1.000000 x,y,z(Bohr):   6.365815 -4.145167   0.167812
69(C) --> Charge: 6.000000 x,y,z(Bohr):   6.612327 -0.072297 -0.020484
70(C) --> Charge: 6.000000 x,y,z(Bohr):   5.146852   2.144572 -0.134370
71(H) --> Charge: 1.000000 x,y,z(Bohr):   6.047010   4.002955 -0.245853
72(C) --> Charge: 6.000000 x,y,z(Bohr):   9.376710 -0.043701 -0.022582
73(C) --> Charge: 6.000000 x,y,z(Bohr): 13.314676   1.433571 -0.021966
74(C) --> Charge: 6.000000 x,y,z(Bohr): 13.276491 -1.251027 -0.020983
75(C) --> Charge: 6.000000 x,y,z(Bohr): 15.566670 -2.645638 -0.000596
76(C) --> Charge: 6.000000 x,y,z(Bohr): 17.787708 -1.197092   0.045519
77(H) --> Charge: 1.000000 x,y,z(Bohr): 19.608379 -2.164164   0.107329
78(C) --> Charge: 6.000000 x,y,z(Bohr): 17.780805   1.461222   0.057583
79(H) --> Charge: 1.000000 x,y,z(Bohr): 19.587982   2.456503   0.075020
80(C) --> Charge: 6.000000 x,y,z(Bohr): 15.545426   2.883143   0.009128
81(C) --> Charge: 6.000000 x,y,z(Bohr): 15.535205   5.683005   0.014800
82(C) --> Charge: 6.000000 x,y,z(Bohr): 17.200196   7.056747   1.570614
83(H) --> Charge: 1.000000 x,y,z(Bohr): 18.486285   6.043270   2.827133
84(C) --> Charge: 6.000000 x,y,z(Bohr): 17.199773   9.689452   1.566656
85(H) --> Charge: 1.000000 x,y,z(Bohr): 18.516088 10.668473   2.817870
86(C) --> Charge: 6.000000 x,y,z(Bohr): 15.546429 11.093465   0.021830
87(C) --> Charge: 6.000000 x,y,z(Bohr): 13.898063   9.714669 -1.532289
88(H) --> Charge: 1.000000 x,y,z(Bohr): 12.588157 10.686333 -2.788738
89(C) --> Charge: 6.000000 x,y,z(Bohr): 13.890979   7.071145 -1.541140
90(H) --> Charge: 1.000000 x,y,z(Bohr): 12.618890   6.083846 -2.833340
91(C) --> Charge: 6.000000 x,y,z(Bohr): 15.604608 13.998596   0.092912
92(C) --> Charge: 6.000000 x,y,z(Bohr): 18.267769 14.929597 -0.665288
93(H) --> Charge: 1.000000 x,y,z(Bohr): 19.734914 14.212687   0.623591
94(H) --> Charge: 1.000000 x,y,z(Bohr): 18.343321 17.011144 -0.615933
95(H) --> Charge: 1.000000 x,y,z(Bohr): 18.747596 14.308486 -2.594398
96(C) --> Charge: 6.000000 x,y,z(Bohr): 14.993837 14.907428   2.801579
97(H) --> Charge: 1.000000 x,y,z(Bohr): 13.099775 14.269095   3.386865
98(H) --> Charge: 1.000000 x,y,z(Bohr): 15.032861 16.988779   2.888288
99(H) --> Charge: 1.000000 x,y,z(Bohr): 16.369730 14.190362   4.187435
100(C) --> Charge: 6.000000 x,y,z(Bohr): 13.670605 15.185889 -1.725350
101(H) --> Charge: 1.000000 x,y,z(Bohr): 14.036878 14.648228 -3.702611
102(H) --> Charge: 1.000000 x,y,z(Bohr): 13.782832 17.260635 -1.606356
103(H) --> Charge: 1.000000 x,y,z(Bohr): 11.721122 14.632843 -1.249483
104(C) --> Charge: 6.000000 x,y,z(Bohr): 15.642956 -5.445399 -0.028319
105(C) --> Charge: 6.000000 x,y,z(Bohr): 13.838642 -6.913546   1.255014
106(H) --> Charge: 1.000000 x,y,z(Bohr): 12.316096 -5.977246   2.276497
107(C) --> Charge: 6.000000 x,y,z(Bohr): 13.965715 -9.552853   1.252092
108(H) --> Charge: 1.000000 x,y,z(Bohr): 12.520344 -10.586575   2.293039
109(C) --> Charge: 6.000000 x,y,z(Bohr): 15.884764 -10.857572 -0.034054
110(C) --> Charge: 6.000000 x,y,z(Bohr): 17.680931 -9.377896 -1.326384

111(H) --> Charge: 1.000000 x,y,z(Bohr): 19.203681 -10.292770 -2.376156
112(C) --> Charge: 6.000000 x,y,z(Bohr): 17.569456 -6.747378 -1.328019
113(H) --> Charge: 1.000000 x,y,z(Bohr): 18.981478 -5.685363 -2.394632
114(C) --> Charge: 6.000000 x,y,z(Bohr): 16.079684 -13.757205 -0.084797
115(C) --> Charge: 6.000000 x,y,z(Bohr): 13.954980 -15.033629 1.435919
116(H) --> Charge: 1.000000 x,y,z(Bohr): 12.073864 -14.566720 0.676977
117(H) --> Charge: 1.000000 x,y,z(Bohr): 14.002681 -14.482600 3.442623
118(H) --> Charge: 1.000000 x,y,z(Bohr): 14.175265 -17.101269 1.345040
119(C) --> Charge: 6.000000 x,y,z(Bohr): 18.641461 -14.572554 1.058489
120(H) --> Charge: 1.000000 x,y,z(Bohr): 18.815749 -16.648764 1.029886
121(H) --> Charge: 1.000000 x,y,z(Bohr): 18.806250 -13.934974 3.034398
122(H) --> Charge: 1.000000 x,y,z(Bohr): 20.247845 -13.787680 -0.005182
123(C) --> Charge: 6.000000 x,y,z(Bohr): -17.433153 -15.186134 -1.725437
124(H) --> Charge: 1.000000 x,y,z(Bohr): -17.317638 -17.260898 -1.609870
125(H) --> Charge: 1.000000 x,y,z(Bohr): -17.060981 -14.644010 -3.700365
126(H) --> Charge: 1.000000 x,y,z(Bohr): -19.385262 -14.637506 -1.255331
127(C) --> Charge: 6.000000 x,y,z(Bohr): -16.126874 -14.912973 2.806703
128(H) --> Charge: 1.000000 x,y,z(Bohr): -18.023554 -14.277534 3.386536
129(H) --> Charge: 1.000000 x,y,z(Bohr): -14.756527 -14.197376 4.198790
130(H) --> Charge: 1.000000 x,y,z(Bohr): -16.086101 -16.994430 2.889821
131(C) --> Charge: 6.000000 x,y,z(Bohr): -12.840264 -14.925088 -0.648089
132(H) --> Charge: 1.000000 x,y,z(Bohr): -12.761645 -17.006602 -0.601616
133(H) --> Charge: 1.000000 x,y,z(Bohr): -11.379110 -14.207584 0.647304
134(H) --> Charge: 1.000000 x,y,z(Bohr): -12.354176 -14.300314 -2.574462

Optimized structure - at C-PCM(DMF)/B3LYP(D3)/cc-pVTZ level of theory – of the Stop-[Bzi-Bipy-Bzi]-Stop molecular thread in the *cis* conformation (see Figure 3, right panel in the manuscript):

1(N) --> Charge: 7.000000 x,y,z(Bohr): 2.564224 1.557326 -0.571263
2(N) --> Charge: 7.000000 x,y,z(Bohr): -2.575047 1.541258 0.419379
3(N) --> Charge: 7.000000 x,y,z(Bohr): -10.593249 1.998968 0.035293
4(N) --> Charge: 7.000000 x,y,z(Bohr): -11.037287 -2.203021 -0.278240
5(H) --> Charge: 1.000000 x,y,z(Bohr): -10.573960 -4.058589 -0.223015
6(N) --> Charge: 7.000000 x,y,z(Bohr): 10.723631 1.848729 -0.157724
7(H) --> Charge: 1.000000 x,y,z(Bohr): 10.004674 3.621061 -0.118012
8(N) --> Charge: 7.000000 x,y,z(Bohr): 10.884175 -2.375354 0.122207
9(C) --> Charge: 6.000000 x,y,z(Bohr): 1.396123 -0.665571 -0.137765
10(C) --> Charge: 6.000000 x,y,z(Bohr): 2.764030 -2.895179 0.352708
11(H) --> Charge: 1.000000 x,y,z(Bohr): 1.789091 -4.666442 0.746858
12(C) --> Charge: 6.000000 x,y,z(Bohr): 5.386718 -2.814928 0.394914
13(H) --> Charge: 1.000000 x,y,z(Bohr): 6.499796 -4.503713 0.786024
14(C) --> Charge: 6.000000 x,y,z(Bohr): 6.621228 -0.507731 -0.054213
15(C) --> Charge: 6.000000 x,y,z(Bohr): 5.083015 1.608284 -0.536052
16(H) --> Charge: 1.000000 x,y,z(Bohr): 5.929407 3.451930 -0.941478
17(C) --> Charge: 6.000000 x,y,z(Bohr): 9.383167 -0.378009 -0.022081
18(C) --> Charge: 6.000000 x,y,z(Bohr): 13.262224 1.246043 -0.080121
19(C) --> Charge: 6.000000 x,y,z(Bohr): 13.324157 -1.431352 0.107917
20(C) --> Charge: 6.000000 x,y,z(Bohr): 15.663795 -2.733029 0.247652
21(C) --> Charge: 6.000000 x,y,z(Bohr): 17.829047 -1.202142 0.214935
22(H) --> Charge: 1.000000 x,y,z(Bohr): 19.683288 -2.093669 0.360105
23(C) --> Charge: 6.000000 x,y,z(Bohr): 17.723112 1.448464 0.043542
24(H) --> Charge: 1.000000 x,y,z(Bohr): 19.491448 2.510760 0.009113
25(C) --> Charge: 6.000000 x,y,z(Bohr): 15.437118 2.778042 -0.124118
26(C) --> Charge: 6.000000 x,y,z(Bohr): 15.322764 5.569493 -0.309363

27(C) --> Charge: 6.000000 x,y,z(Bohr): 16.912559 7.107321 1.156913
28(H) --> Charge: 1.000000 x,y,z(Bohr): 18.222914 6.233893 2.491214
29(C) --> Charge: 6.000000 x,y,z(Bohr): 16.817960 9.741495 0.977026
30(H) --> Charge: 1.000000 x,y,z(Bohr): 18.087854 10.836475 2.171951
31(C) --> Charge: 6.000000 x,y,z(Bohr): 15.133259 10.964628 -0.668770
32(C) --> Charge: 6.000000 x,y,z(Bohr): 13.550523 9.412378 -2.140871
33(H) --> Charge: 1.000000 x,y,z(Bohr): 12.221137 10.263201 -3.469312
34(C) --> Charge: 6.000000 x,y,z(Bohr): 13.637933 6.784859 -1.974761
35(H) --> Charge: 1.000000 x,y,z(Bohr): 12.417337 5.662088 -3.204386
36(C) --> Charge: 6.000000 x,y,z(Bohr): 14.964903 13.856453 -0.909479
37(C) --> Charge: 6.000000 x,y,z(Bohr): 12.262023 14.724515 -0.219660
38(H) --> Charge: 1.000000 x,y,z(Bohr): 11.792647 14.196881 1.739642
39(H) --> Charge: 1.000000 x,y,z(Bohr): 10.833609 13.876954 -1.472347
40(H) --> Charge: 1.000000 x,y,z(Bohr): 12.105619 16.795202 -0.389494
41(C) --> Charge: 6.000000 x,y,z(Bohr): 16.838216 15.220954 0.846826
42(H) --> Charge: 1.000000 x,y,z(Bohr): 16.646653 17.279468 0.607675
43(H) --> Charge: 1.000000 x,y,z(Bohr): 18.810927 14.717745 0.413349
44(H) --> Charge: 1.000000 x,y,z(Bohr): 16.478646 14.783992 2.849929
45(C) --> Charge: 6.000000 x,y,z(Bohr): 15.559518 14.631424 -3.662922
46(H) --> Charge: 1.000000 x,y,z(Bohr): 14.222530 13.783338 -5.012425
47(H) --> Charge: 1.000000 x,y,z(Bohr): 17.481117 14.035239 -4.200944
48(H) --> Charge: 1.000000 x,y,z(Bohr): 15.440406 16.701245 -3.869386
49(C) --> Charge: 6.000000 x,y,z(Bohr): 15.843065 -5.523156 0.414149
50(C) --> Charge: 6.000000 x,y,z(Bohr): 17.825003 -6.839336 -0.783544
51(H) --> Charge: 1.000000 x,y,z(Bohr): 19.205080 -5.801471 -1.913900
52(C) --> Charge: 6.000000 x,y,z(Bohr): 18.031686 -9.457707 -0.600164
53(H) --> Charge: 1.000000 x,y,z(Bohr): 19.593702 -10.386271 -1.577694
54(C) --> Charge: 6.000000 x,y,z(Bohr): 16.281361 -10.909870 0.783013
55(C) --> Charge: 6.000000 x,y,z(Bohr): 14.307318 -9.591208 1.967857
56(H) --> Charge: 1.000000 x,y,z(Bohr): 12.893679 -10.603296 3.071744
57(C) --> Charge: 6.000000 x,y,z(Bohr): 14.084162 -6.964446 1.787544
58(H) --> Charge: 1.000000 x,y,z(Bohr): 12.522266 -6.016169 2.736086
59(C) --> Charge: 6.000000 x,y,z(Bohr): 16.580942 -13.797242 0.931470
60(C) --> Charge: 6.000000 x,y,z(Bohr): 19.164971 -14.439875 2.133218
61(H) --> Charge: 1.000000 x,y,z(Bohr): 20.747243 -13.671585 1.022356
62(H) --> Charge: 1.000000 x,y,z(Bohr): 19.413790 -16.505576 2.246152
63(H) --> Charge: 1.000000 x,y,z(Bohr): 19.297472 -13.664161 4.061583
64(C) --> Charge: 6.000000 x,y,z(Bohr): 16.460755 -14.919335 -1.763383
65(H) --> Charge: 1.000000 x,y,z(Bohr): 14.631821 -14.490042 -2.662597
66(H) --> Charge: 1.000000 x,y,z(Bohr): 16.678685 -16.990431 -1.692530
67(H) --> Charge: 1.000000 x,y,z(Bohr): 17.968207 -14.165243 -2.982801
68(C) --> Charge: 6.000000 x,y,z(Bohr): 14.496522 -15.043081 2.531213
69(H) --> Charge: 1.000000 x,y,z(Bohr): 14.514618 -14.354459 4.495472
70(H) --> Charge: 1.000000 x,y,z(Bohr): 14.791965 -17.102706 2.582849
71(H) --> Charge: 1.000000 x,y,z(Bohr): 12.603405 -14.698280 1.737631
72(C) --> Charge: 6.000000 x,y,z(Bohr): -1.420033 -0.654499 -0.182035
73(C) --> Charge: 6.000000 x,y,z(Bohr): -2.799583 -2.828159 -0.838948
74(H) --> Charge: 1.000000 x,y,z(Bohr): -1.837939 -4.564167 -1.389400
75(C) --> Charge: 6.000000 x,y,z(Bohr): -5.427487 -2.736359 -0.851937
76(H) --> Charge: 1.000000 x,y,z(Bohr): -6.496438 -4.409441 -1.410644
77(C) --> Charge: 6.000000 x,y,z(Bohr): -6.644309 -0.473586 -0.197450
78(C) --> Charge: 6.000000 x,y,z(Bohr): -5.089797 1.603747 0.410733
79(H) --> Charge: 1.000000 x,y,z(Bohr): -5.979556 3.396120 0.923395
80(C) --> Charge: 6.000000 x,y,z(Bohr): -9.393392 -0.190149 -0.142691

81(C) --> Charge: 6.000000 x,y,z(Bohr): -13.463469 -1.247823 -0.156493
82(C) --> Charge: 6.000000 x,y,z(Bohr): -13.143101   1.410742   0.033360
83(C) --> Charge: 6.000000 x,y,z(Bohr): -15.269755   3.027187   0.261624
84(C) --> Charge: 6.000000 x,y,z(Bohr): -17.628032   1.814255   0.309161
85(H) --> Charge: 1.000000 x,y,z(Bohr): -19.337527   2.962018   0.427219
86(C) --> Charge: 6.000000 x,y,z(Bohr): -17.900911 -0.823844   0.132568
87(H) --> Charge: 1.000000 x,y,z(Bohr): -19.802562 -1.623962   0.128562
88(C) --> Charge: 6.000000 x,y,z(Bohr): -15.831566 -2.460199 -0.121279
89(C) --> Charge: 6.000000 x,y,z(Bohr): -16.116685 -5.237801 -0.322184
90(C) --> Charge: 6.000000 x,y,z(Bohr): -14.658567 -6.668278 -2.019295
91(H) --> Charge: 1.000000 x,y,z(Bohr): -13.313491 -5.724405 -3.269155
92(C) --> Charge: 6.000000 x,y,z(Bohr): -14.945214 -9.289622 -2.203924
93(H) --> Charge: 1.000000 x,y,z(Bohr): -13.769673 -10.294271 -3.563141
94(C) --> Charge: 6.000000 x,y,z(Bohr): -16.700685 -10.603186 -0.711707
95(C) --> Charge: 6.000000 x,y,z(Bohr): -18.165600 -9.155681   0.976404
96(H) --> Charge: 1.000000 x,y,z(Bohr): -19.554132 -10.084142   2.187406
97(C) --> Charge: 6.000000 x,y,z(Bohr): -17.886734 -6.545321   1.173898
98(H) --> Charge: 1.000000 x,y,z(Bohr): -19.033338 -5.502534   2.536886
99(C) --> Charge: 6.000000 x,y,z(Bohr): -17.069126 -13.482662 -0.856379
100(C) --> Charge: 6.000000 x,y,z(Bohr): -19.825627 -14.062755 -1.630970
101(H) --> Charge: 1.000000 x,y,z(Bohr): -20.125546 -16.121876 -1.736598
102(H) --> Charge: 1.000000 x,y,z(Bohr): -20.256762 -13.247542 -3.498321
103(H) --> Charge: 1.000000 x,y,z(Bohr): -21.191827 -13.293401 -0.263647
104(C) --> Charge: 6.000000 x,y,z(Bohr): -16.530386 -14.657456   1.763697
105(H) --> Charge: 1.000000 x,y,z(Bohr): -17.806461 -13.908076   3.225831
106(H) --> Charge: 1.000000 x,y,z(Bohr): -14.572555 -14.271370   2.359804
107(H) --> Charge: 1.000000 x,y,z(Bohr): -16.792049 -16.723237   1.693122
108(C) --> Charge: 6.000000 x,y,z(Bohr): -15.294464 -14.726004 -2.795601
109(H) --> Charge: 1.000000 x,y,z(Bohr): -13.291751 -14.416890 -2.319749
110(H) --> Charge: 1.000000 x,y,z(Bohr): -15.626176 -14.006356 -4.720359
111(H) --> Charge: 1.000000 x,y,z(Bohr): -15.624311 -16.780634 -2.827046
112(C) --> Charge: 6.000000 x,y,z(Bohr): -15.044103   5.811531   0.458662
113(C) --> Charge: 6.000000 x,y,z(Bohr): -16.733298   7.220830   1.947848
114(H) --> Charge: 1.000000 x,y,z(Bohr): -18.188409   6.248391   3.041716
115(C) --> Charge: 6.000000 x,y,z(Bohr): -16.563657   9.852595   2.109516
116(H) --> Charge: 1.000000 x,y,z(Bohr): -17.920014 10.835323   3.307412
117(C) --> Charge: 6.000000 x,y,z(Bohr): -14.699972 11.207314   0.794569
118(C) --> Charge: 6.000000 x,y,z(Bohr): -13.006810   9.785405 -0.688326
119(H) --> Charge: 1.000000 x,y,z(Bohr): -11.522039 10.744288 -1.753581
120(C) --> Charge: 6.000000 x,y,z(Bohr): -13.161135   7.162367 -0.855028
121(H) --> Charge: 1.000000 x,y,z(Bohr): -11.807652   6.136341 -2.018629
122(C) --> Charge: 6.000000 x,y,z(Bohr): -14.447550 14.099937   0.924316
123(C) --> Charge: 6.000000 x,y,z(Bohr): -16.467181 15.309630   2.631276
124(C) --> Charge: 6.000000 x,y,z(Bohr): -14.731718 15.215033 -1.761064
125(H) --> Charge: 1.000000 x,y,z(Bohr): -13.278606 14.486130 -3.059098
126(H) --> Charge: 1.000000 x,y,z(Bohr): -14.546917 17.289704 -1.703010
127(C) --> Charge: 6.000000 x,y,z(Bohr): -11.815581 14.792466   1.986428
128(H) --> Charge: 1.000000 x,y,z(Bohr): -11.599290 16.862634   2.084380
129(H) --> Charge: 1.000000 x,y,z(Bohr): -10.281058 14.050936   0.793457
130(H) --> Charge: 1.000000 x,y,z(Bohr): -11.566462 14.022671   3.905604
131(H) --> Charge: 1.000000 x,y,z(Bohr): -16.598301 14.752269 -2.560795
132(H) --> Charge: 1.000000 x,y,z(Bohr): -16.212833 17.375184   2.659360
133(H) --> Charge: 1.000000 x,y,z(Bohr): -18.393064 14.921274   1.943537
134(H) --> Charge: 1.000000 x,y,z(Bohr): -16.327354 14.631460   4.594248

Local *H(r)=G(r)+V(r)* analysis in the presence of Zn(II) cation:

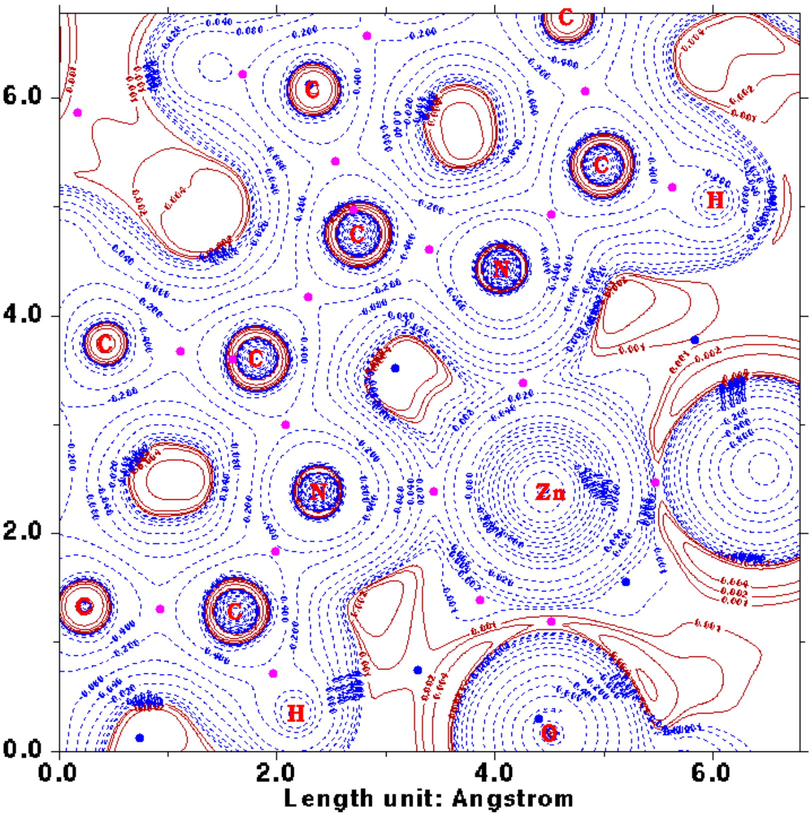

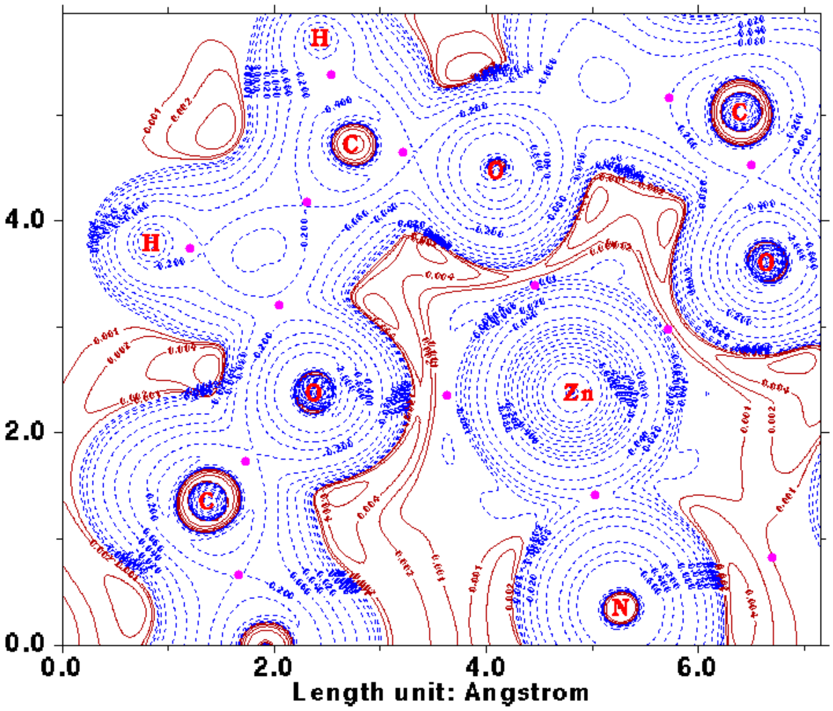

Figure S5. Figure 4a and Figure 4b with the default - from MultiWfn program, see ref. 36 into the associated manuscript - contour lines showing the associated *H(r)=G(r)+V(r)* value for the investigated [2]rotaxane in the presence of Zn(II) computed at C-PCM-B3LYP(D3)/cc-pVTZ level of theory.

Conceptual DFT (CDFT) analysis:

Local reactivity descriptors for the two investigated molecular threads are also addressed in the field of the Conceptual DFT (CDFT) framework.[1-4] In particular, we have characterized electrophilic and nucleophilic regions potentially prone to complexation pathways by using the empirically defined Orbital-Weighted Fukui functions [$f_w^-(\boldsymbol{r})$ and $f_w^+(\boldsymbol{r})$][5] as defined by the following expressions:

$$f_w^-(\boldsymbol{r}) = \sum_{i}^{HOMO} w_i \rho_i(\boldsymbol{r}) \;\; ; \; f_w^+(\boldsymbol{r}) = \sum_{LUMO}^{\infty} w_i \rho_i(\boldsymbol{r})$$

in which w_i are the so-called weighting factors, $\rho_i(\boldsymbol{r})$ represents the square of the *i*-th molecular orbital and the term *i* runs over a set of MOs. The w_i terms are defined - for both the $f_w^-(\boldsymbol{r})$ and $f_w^+(\boldsymbol{r})$ functions - according to the Gaussian ansatz as follows:

$$w_i = \frac{\exp\left[-\left(\frac{\mu-\epsilon_i}{\Delta}\right)^2\right]}{\sum_i^{HOMO} \exp\left[-\left(\frac{\mu-\epsilon_i}{\Delta}\right)^2\right]} \;\; ; \; w_i = \frac{\exp\left[-\left(\frac{\mu-\epsilon_i}{\Delta}\right)^2\right]}{\sum_{LUMO}^{\infty} \exp\left[-\left(\frac{\mu-\epsilon_i}{\Delta}\right)^2\right]}$$

where μ is the chemical potential - approximately estimated as (E_{HOMO}+E_{LUMO})/2) - ϵ_i the energy of each molecular orbital and Δ represents the width of a Gaussian-type function determining the energy range for orbitals that make a substantial contribution to the reactivity. A Gaussian model was chosen, as a matter of convenience, because the numbers of molecular orbitals and its relative contribution to reactivity can be controlled with a single parameter, Δ, typically set as first guess to 0.1 Hartree.[5] We applied the formulations reported above to closed-shell single-determinant wavefunctions taking into account the functionalized Stop-[Bzi-Bipy-Bzi]-Stop molecular thread in its *cis* and *trans* conformations (see also Figure 3 into the manuscript). Moreover, computations of CDFT descriptors are done by executing the Multiwfn program (version 3.8.dev); [6] in this context, we do not consider diffuse functions to avoid spurious effects in the definition of $f_w^-(\boldsymbol{r})$ and $f_w^+(\boldsymbol{r})$ functions. Please note that we recently used the same approach over a similar system in aqueous solution.[7]

In the Figure S6 we report the plot of the $f_w^-(\boldsymbol{r})$ Orbital-Weighted Fukui functions for the *cis* conformation of the Stop-[Bzi-Bipy-Bzi]-Stop molecular thread according to its mathematical definition reported above; for the sake of completeness, in this figure the condensed OW Fukui indices are also displayed for the atomic species showing the largest values characterizing the *cis* and *trans* conformations, respectively. Looking at this figure, we can easily realize that, in both the investigated structures, the unsaturated nitrogen atom of the Bzi and Bipy moieties feature the most relevant site prone for an electrophilic attack by cationic species. Local condensed value of $f_w^-(\boldsymbol{r})$ in the range between 0.0226-0.0271 $e \cdot a_0^{-3}$ are estimated from converged electronic wavefunction at C-PCM/B3LYP level of computation for the *trans* conformation (upper left panel in Fig. S6). In line with this, the *cis* conformation of the Stop-[Bzi-Bipy-Bzi]-Stop molecular thread clearly

shows the nucleophilic properties of the Bipy chelating moieties that play a crucial role for Zn(II) and Pt(II) cations attachment as observed by Loeb and co-workers (see ref. 21 in the manuscript). More specifically, the cation-chelating site featuring values of $f_w^-(\boldsymbol{r})$ lying at 0.0252 and 0.0247 $e{\cdot}a_0^{-3}$ (which provides an estimation of the nucleophilicity of the unsaturated N atoms, see Fig. S6) reflects the non-symmetric electronic distribution arising from the absence of co-planarity in the *cis* form of the Bipy central unit. This trend further supports the observations made by analyzing the Electrostatic Potential (ESP) surface for the same structures (see Figure 3 in the manuscript).

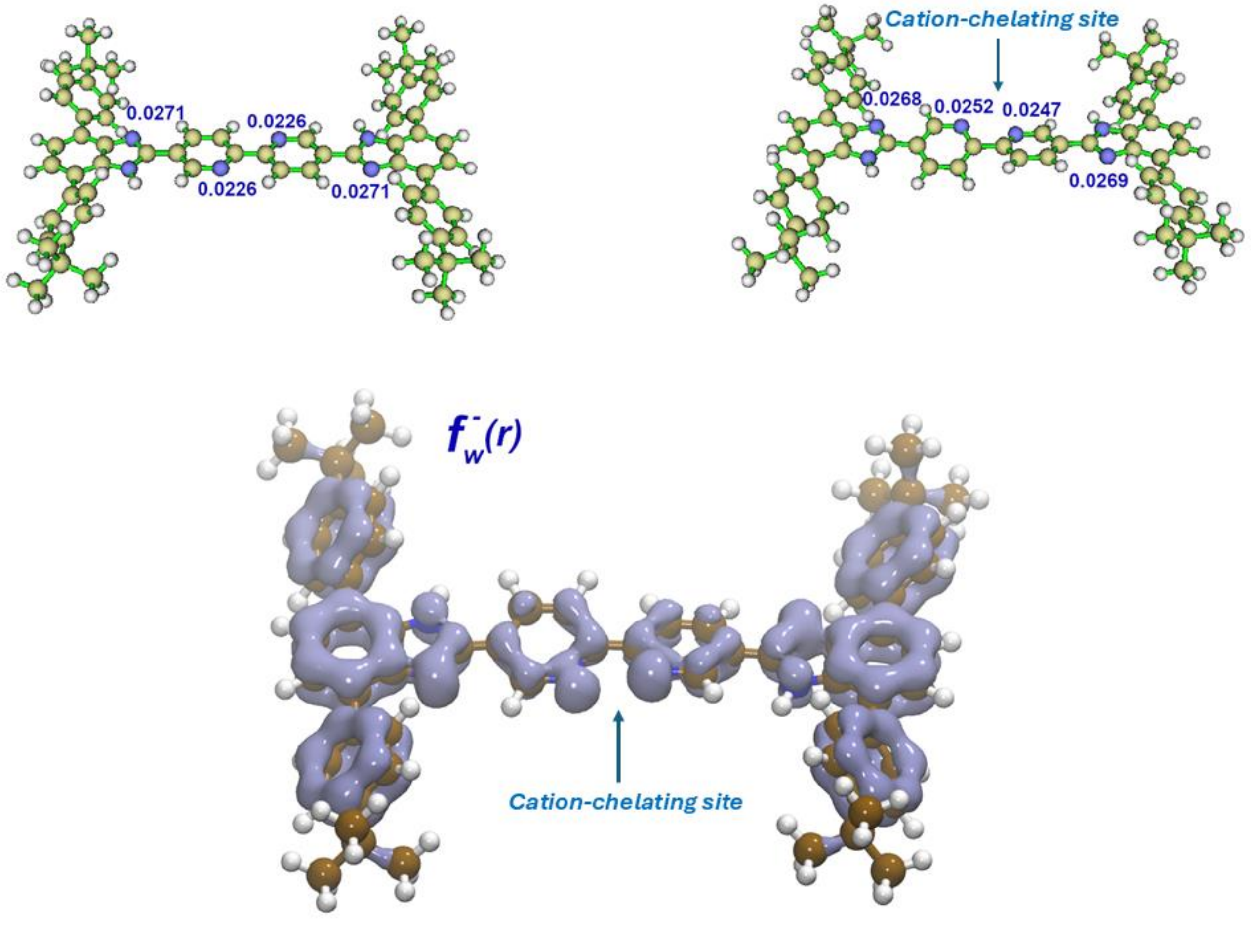

Figure S6. Orbital-Weighted condensed Fukui indices for the *cis* and *trans* conformations of the investigated Stop-[Bzi-Bipy-Bzi]-Stop molecular thread at C-PCM(DMF)-B3LYP(D3)/cc-pVTZ level of theory; the 3D plot of the $f^-_w(\mathbf{r})$ function for the *cis* form of the two investigated thread is also displayed with an isovalue of 0.0005 $e{\cdot}a_0^{-3}$.

PBE0 Computations

As explained in the text of the manuscript, we also applied a parameter free density functional (the PBE0) obtained by combining the PBE generalized gradient functional with a predefinite amount of exact exchange at Hartree-Fock level. In particular we focused our attention on the electronic and structural properties derived applying the PBE0 functional on the transient species at the mid-point along the shuttling process of

a 24C8 macrocycle between the two Bzi recognition sites in the presence of the Zn(II) cation over the Bipy unit. More specifically, the optimized geometry derived at C-PCM(DMF)-PBE0(D3)/cc-pVTZ level of theory has been compared with that previously estimated at C-PCM(DMF)-B3LYP(D3)/cc-pVTZ level. As it can be appreciated from Figure S7, the two applied hybrid and GGA-based functionals provide very similar geometrical structures for the [2]rotaxane in the presence of Zn(II). This trend actually reinforces the analyses and the correlated conclusions reported in the text of the manuscript while addressing - with an accurate level of theory – a plausible octahedral intermediate compound originally proposed by Loeb and co-workers via PCM(DMF)-B3LYP-D3/STO-3G* simulations (see ref. 21 into the manuscript for major details).

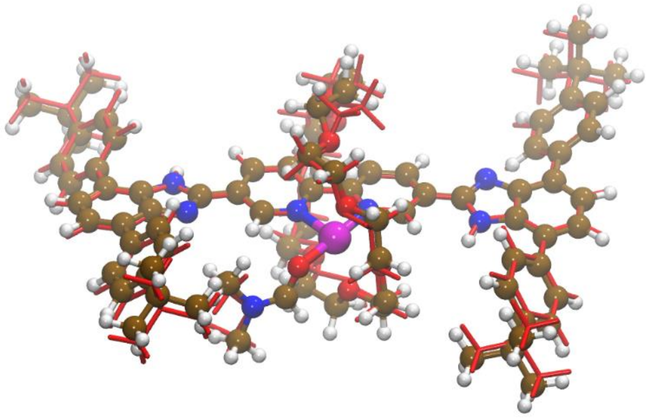

Figure S7. Superimposing of the C-PCM(DMF)/B3LYP(D3)/cc-pVTZ *vs* C-PCM(DMF)/PBE0(D3)/cc-pVTZ optimized structures characterizing a plausible octahedral intermediate compound at the midpoint along the shuttling process involving the 24C8 macrocycle over a symmetric Bzi-Bipy-Bzi molecular thread in the presence of Zn(II) cation. The computed PBE0 structure is shown using red color.

Moreover, a direct comparison between the two optimized structures is also analyzed in terms of QTAIM indicators (see Table 2 in the associated manuscript with data collected at C-CPM/B3LYP level). More in detail, for the local chemical contacts modulating the complexation between the Zn(II) cation and the 2[rotaxane] + DMF molecule stabilizing such a transient species in DMF solvent, we can appreciate that B3LYP and PBE0 functionals provides substantially the same interaction scenario:

Table S2. Electron density $\rho(\mathbf{r})$ ($e \cdot a_0^{-3}$), Laplacian of electron density $\nabla^2\rho(\mathbf{r})$ ($e \cdot a_0^{-5}$), electron kinetic energy density $G(\mathbf{r})$ (hartree$\cdot a_0^{-3}$), electron potential energy density $V(\mathbf{r})$ (hartree$\cdot a_0^{-3}$), and electron energy density $H(\mathbf{r})$ (hartree$\cdot a_0^{-3}$) for bond critical points on selected bonds of the optimized [2]molecular shuttle with Zn(II) calculated at C-PCM(DMF)/[B3LYP(D3)]*vs*[PBE0(D3)]/cc-pVTZ level of theory.

BCP	Moieties	Distance (Å)	$\rho(\mathbf{r})$	$\nabla^2\rho(\mathbf{r})$	$G(\mathbf{r})$	$V(\mathbf{r})$	$-G(\mathbf{r})/V(\mathbf{r})$	$H(\mathbf{r})$
**B3LYP**								
Zn(II) – O=C (equat.)	Ion-DMF	2.02	0.0703	0.3862	0.1011	-0.1057	0.9567	-0.0046
Zn(II) – N (equat.)	Ion-Bipy	2.07	0.0751	0.3254	0.0935	-0.1056	0.8854	-0.0121
Zn(II) – N (equat.)	Ion-Bipy	2.09	0.0720	0.3106	0.0882	-0.0987	0.8933	-0.0105
Zn(II) – O (equat.)	Ion-Mac	2.20	0.0465	0.2084	0.0529	-0.0537	0.9847	-0.0008
Zn(II) – O (axial)	Ion-Mac	2.27	0.0448	0.2127	0.0529	-0.0534	0.9904	-0.0005

Zn(II) – O (axial)	Ion-Mac	2.33	0.0293	0.1093	0.0277	-0.0280	0.9876	-0.0003
**PBE0**								
Zn(II) – O=C (equat.)	Ion-DMF	2.02	0.0701	0.3883	0.1018	-0.1066	0.9550	-0.0048
Zn(II) – N (equat.)	Ion-Bipy	2.06	0.0761	0.3336	0.0963	-0.1092	0.8819	-0.0129
Zn(II) – N (equat.)	Ion-Bipy	2.08	0.0738	0.3221	0.0923	-0.1040	0.8875	-0.0117
Zn(II) – O (equat.)	Ion-Mac	2.17	0.0516	0.2466	0.0631	-0.0644	0.9798	-0.0013
Zn(II) – O (axial)	Ion-Mac	2.21	0.0497	0.2494	0.0624	-0.0631	0.9889	-0.0007
Zn(II) – O (axial)	Ion-Mac	2.39	0.0259	0.0859	0.0230	-0.0231	0.9957	-0.0001

References:

1 P. Geerlings, F. De Proft, W. Langenaeker, Conceptual Density Functional Theory, *Chem. Rev.*, **2003**, *103*, 1793–1874.

2 P. K. Chattaraj, U. Sarkar, D. R. Roy, Electrophilicity Index*, Chem. Rev*., **2006**, *106*, 2065–2091 and quoted references therein cited.

3 Conceptual Density Functional Theory: Towards a New Chemical Reactivity Theory, Editor(s): Shubin Liu, Wiley-VCH GmbH, **2022**.

4 K. R. S. Chandrakumar, S. Pal, The Concept of Density Functional Theory Based Descriptors and its Relation with the Reactivity of Molecular Systems: A Semi-Quantitative Study, *Int. J. Mol. Sci.*, **2002**, *3*, 324.

5 R. Pino-Rios, O. Yañez, D. Inostroza, L. Ruiz, C. Cardenas, P. Fuentealba, W. Tiznado, Proposal of a simple and effective local reactivity descriptor through a topological analysis of an orbital-weighted fukui function, *J. Comput, Chem.,* **2017**, *38*, 481-488.

6 T. Lu and F. Chen, Multiwfn: A multifunctional wavefunction analyser, *J. Comput. Chem.*, **2012**, *33*, 580-592.

7 C. Zazza, S.Borocci, N. Sanna, F. Grandinetti, On the electronic properties of a pH-responsive molecular thread for H-shaped molecular shuttles in aqueous solution, *Chem. Phys. Lett.*, **2025**, *858*, 141740.
